# Blood-based epigenome-wide analyses of cognitive abilities

**DOI:** 10.1186/s13059-021-02596-5

**Published:** 2022-01-17

**Authors:** Daniel L. McCartney, Robert F. Hillary, Eleanor L. S. Conole, Daniel Trejo Banos, Danni A. Gadd, Rosie M. Walker, Cliff Nangle, Robin Flaig, Archie Campbell, Alison D. Murray, Susana Muñoz Maniega, María del C. Valdés-Hernández, Mathew A. Harris, Mark E. Bastin, Joanna M. Wardlaw, Sarah E. Harris, David J. Porteous, Elliot M. Tucker-Drob, Andrew M. McIntosh, Kathryn L. Evans, Ian J. Deary, Simon R. Cox, Matthew R. Robinson, Riccardo E. Marioni

**Affiliations:** 1 Centre for Genomic and Experimental Medicine, Institute of Genetics and Cancer, University of Edinburgh, Edinburgh, EH4 2XU UK; 2 Lothian Birth Cohorts, Department of Psychology, University of Edinburgh, Edinburgh, EH8 9JZ UK; 3 Centre for Clinical Brain Sciences, UK Dementia Research Institute at the University of Edinburgh, Chancellor’s Building, 49 Little France Crescent, Edinburgh BioQuarter, Edinburgh, EH16 4SB UK; 4 Department of Quantitative Biomedicine, University of Zurich, Zurich, Switzerland; 5 Biomedical Informatics, University Hospital of Zurich, Zurich, Switzerland; 6 Aberdeen Biomedical Imaging Centre, University of Aberdeen, Aberdeen, Scotland, UK; 7 Department of Psychology, University of Texas, Austin, TX USA; 8 Population Research Center and Center on Aging and Population Sciences, University of Texas, Austin, TX USA; 9 Division of Psychiatry, Centre for Clinical Brain Sciences, University of Edinburgh, Edinburgh, UK; 10 Institute of Science and Technology Austria, 3400 Klosterneuburg, Austria

**Keywords:** DNA methylation, EWAS, Cognitive ability, Prediction, Epidemiology

## Abstract

**Background:**

Blood-based markers of cognitive functioning might provide an accessible way to track neurodegeneration years prior to clinical manifestation of cognitive impairment and dementia.

**Results:**

Using blood-based epigenome-wide analyses of general cognitive function, we show that individual differences in DNA methylation (DNAm) explain 35.0% of the variance in general cognitive function (*g*). A DNAm predictor explains ~4% of the variance, independently of a polygenic score, in two external cohorts. It also associates with circulating levels of neurology- and inflammation-related proteins, global brain imaging metrics, and regional cortical volumes.

**Conclusions:**

As sample sizes increase, the ability to assess cognitive function from DNAm data may be informative in settings where cognitive testing is unreliable or unavailable.

**Supplementary Information:**

The online version contains supplementary material available at 10.1186/s13059-021-02596-5.

## Background

Blood-based markers of cognitive functioning might provide an accessible way to track neurodegeneration years prior to clinical manifestation of cognitive impairment and dementia. They might also form an easy, objective, and less stressful way to assess neurodegeneration compared to pen-and-paper cognitive tests or in circumstances where biosamples alone are available. Furthermore, they could help to inform our understanding of the biological basis of brain health differences. Blood-based DNA methylation can be used to generate predictors of lifestyle factors, such as smoking, alcohol consumption, and obesity [1]—factors that are linked with poorer cognitive function and an increased risk of dementia [2]. However, blood-based DNA methylation predictors of cognitive function itself, rather than its known correlates, may index a wider range of risk factors for neurodegeneration. Despite being peripheral to the central nervous system, blood is an easily accessible tissue, and its DNA methylation patterns may enable early diagnosis and provide mechanistic insights of early phases of disease progression. Although DNA methylation in brain tissue may provide more direct insights into the biology of neurodegeneration, [3, 4] acquiring in-vivo brain tissue samples is not feasible outside of extraordinary circumstances. Recent methodological advances [5, 6] have enabled the estimation of variance that DNA methylation can account for in complex traits. Therefore, we can now quantify how well blood-based DNA methylation predicts cognitive test outcomes.

There is modest evidence for associations between individual blood-based methylation sites and cognitive functioning; six CpG probes were identified as genome-wide significant in meta-analyses of epigenome-wide association studies (EWASs) of seven cognitive traits [7]. That study was limited by heterogeneous cognitive outcomes across cohorts, which also varied in age and ethnicity (meta-analysis *n* ranging from 2557 to 6809). Large-scale single cohort studies with consistent cognitive phenotyping and DNA methylation typing and quality control are lacking. Here, we overcome these limitations by utilising phenotypic cognitive data and blood-based methylation data from a single large cohort of European ancestries.

## Results

### The Generation Scotland dataset

Blood-based DNA methylation and general cognitive ability (*g*) were assessed concurrently in 9162 adult participants from the Generation Scotland cohort [8, 9] (Additional file 1, Table S1; Additional file 2, Figure S1; Methods). The study cohort comprised 59% females and had a mean age of 49.8 years (SD 13.6; range 18–93). Prior to running the main analyses, the cognitive phenotypes were pre-corrected for four covariates: age, sex, BMI and an epigenetic smoking score [10]. The DNA methylation data were corrected for batch, age, sex and epigenetic smoking, which all have pervasive effects on DNAm levels. Residuals from these linear regression models were taken forward for the primary analyses.

### Estimating the proportion of variance in cognitive ability explained by all CpG sites

We first explored if global patterns of DNA methylation associated with individual differences in cognitive ability. To determine the proportion of variance in *g* that can be explained by all CpG sites on the DNAm array and to identify individual CpGs associated with *g*, we conducted a Bayesian penalised regression and Gaussian mixture-based variance partitioning analysis using BayesR+ software. BayesR+ has been shown to implicitly control for white cell proportions, which are typically estimated from the DNAm data, related participants, and other unknown confounders [6]. Three mixture distributions were specified, corresponding to possible small, medium and large effect sizes for the CpGs (explaining 0.01%, 0.1% and 1% of the variance, respectively). Variance components analyses indicated that 41.6% [95% credible interval 31.0%, 53.0%] of variance in *g* was explained by all DNA methylation probes (Additional file 1, Table S2).

### Variance component sensitivity analyses

Although BayesR+ can control for genetic relatedness as previously demonstrated in Generation Scotland analyses [6], a sensitivity analysis using a linear mixed model approach [5] with an epigenetic relationship matrix was considered. It yielded near identical results (43.4% (SE 0.03)); a sensitivity analysis using data from an unrelated subset (*n*=4261) of the study cohort that was processed in a single methylation batch also showed similar estimates (58.4% (SE 0.07); Additional file 1, Table S3). Of the prior distributions specified in the BayesR+ analysis, the majority of the variance explained by the DNAm array was accounted for by CpG sites assigned to the mixture corresponding to small effects (Additional file 1, Table S4).

### Estimating the proportion of variance in cognitive ability explained by common SNPs

Previous studies, including those using data from Generation Scotland [11], have identified non-zero common-SNP-based heritability estimates for general cognitive ability. To assess the genetic contribution to variance in *g* and to see if this overlaps with the DNAm component, two additional BayesR+ models were run. The first included genetic data alone, which estimated a SNP-based heritability of 37.9% [18.3%, 52.9%], which is in line with previous GREML estimates from the cohort [11]. The second model considered the proportion of variance explained when combining the effects of genetics and DNAm, resulting in an estimate of 63.8% [50.0%, 73.5%] (Additional file 1, Table S2). Notably, the CpG contribution to the variance accounted for was largely independent of the genetic component—absolute attenuation 6.6% (relative attenuation 15.9%) to the epigenetic effect size estimate in the model that included genetics (estimate 35.0% [24.8%, 46.7%]).

### Epigenome-wide association study to identify individual CpG sites associated with cognitive ability

After identifying a substantial DNAm-based variance component for *g*, we carried out an epigenome-wide association study in BayesR+. We investigated the associations between *g* and individual CpG sites, which were assigned to one of the three mixtures. We identified three unique lead DNAm sites with a posterior inclusion probability (PIP) greater than 0.80 and, after accounting for highly correlated CpG clusters, a group-based PIP>0.95 (Additional file 2, Figure S2; Additional file 1, Table S5; the entire output is available at 10.5281/zenodo.5794029 [12]). For the three lead CpG sites, we queried the EWAS catalog (accessed on April 5, 2021) for associations with other traits at a previously defined epigenome-wide significance threshold of *P*<3.6x10^-8^ (Additional file 1, Table S6) [13, 14]. They have been linked to age, sex, metabolite levels and lung function. Of 28 CpGs identified in previous blood-based EWAS analyses of cognitive ability and Alzheimer’s disease [4, 7, 15], 24 were available for lookup in the present dataset; Generation Scotland data was not included in any of these studies. There was no evidence for replication (maximum PIP of 0.03; Additional file 1, Table S7).

### Epigenetic Score (EpiScore) for cognitive ability tested in two independent cohorts: the Lothian Birth Cohorts of 1921 and 1936

**Fig. 1:**
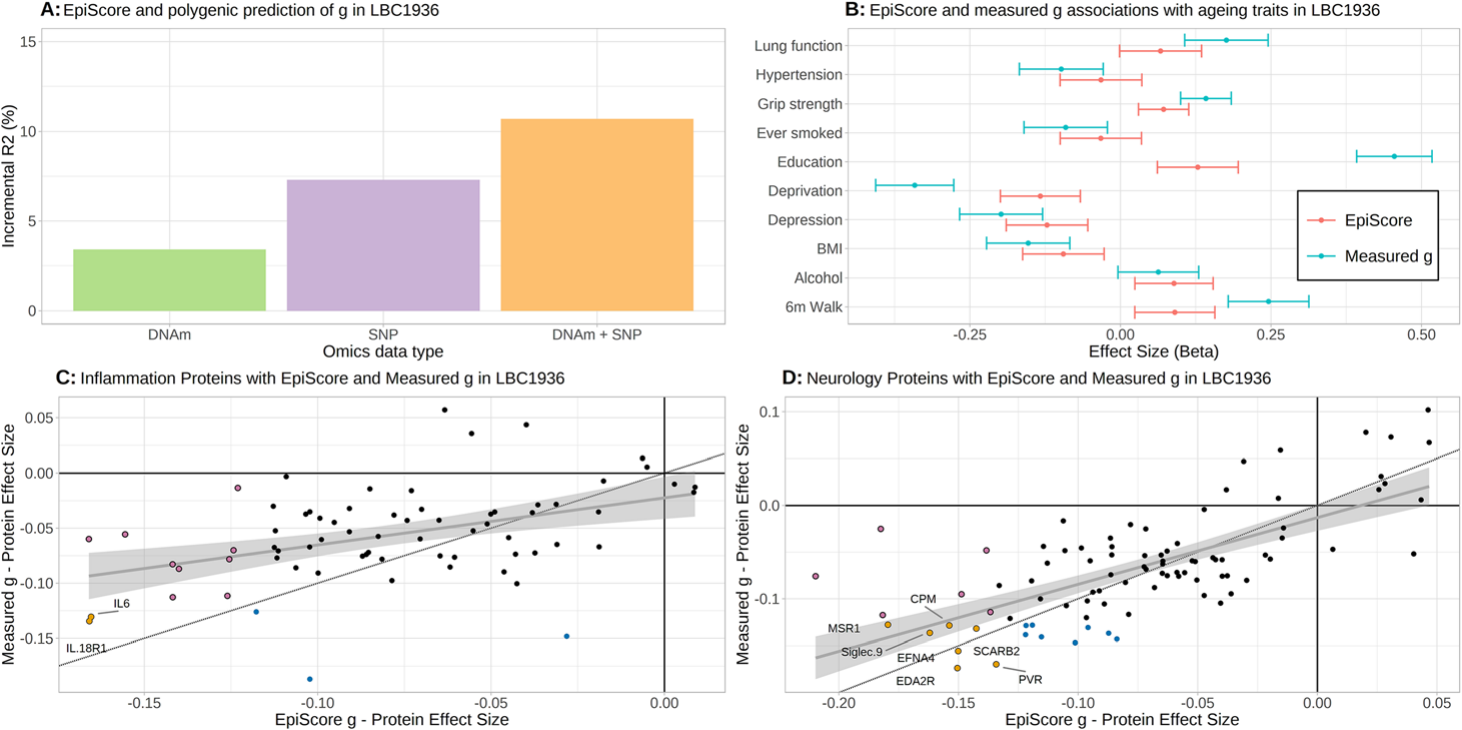
An epigenetic score for cognitive ability associates with measured cognitive ability, health and lifestyle factors and neuro-inflammatory protein levels. Variance explained for general cognitive ability (*g*) by a cognitive Epigenetic Score (EpiScore; green), polygenic score (purple) and in combination (orange) (**A**). Age- and sex-adjusted associations between risk factors for cognitive decline and dementia and the EpiScore (red) and measured *g* (turquoise) with 95% confidence intervals—deprivation and 6-m walk have been reverse coded such that higher values correspond to less deprivation and faster walking speed (**B**). Comparison of age- and sex-adjusted associations between the EpiScore and measured *g* score with 70 inflammation-related (**C**) and 90 neurology-related (**D**) proteins. Coloured points in **C** and **D** are significant after Bonferroni-correction: orange—common to both, pink—unique to EpiScore, blue—unique to measured *g*; dashed lines show perfect correlation (*y*=*x*)—the grey lines show the linear regression slope with 95% confidence interval

A weighted linear Epigenetic Score (EpiScore) for *g* that included all CpG sites in the EWAS was applied to two independent cohorts to determine the proportion of variance in *g* that can be explained by a single predictor variable. Such a predictor may improve risk prediction and patient stratification for studies of cognitive decline and dementia. The weights for each CpG were the mean posterior effect sizes from the EWAS model of *g*. These weights were applied to CpGs in two independent studies, The Lothian Birth Cohort 1936 (LBC1936) and The Lothian Birth Cohort 1921 (LBC1921); *n*=844 and *n*=427 with concurrently measured DNAm, cognitive scores, and cognitive polygenic scores available, respectively (Additional file 1, Table S8; Additional file 2, Figure S3; Methods). The resulting cognitive EpiScores showed very weak correlations (absolute Pearson *r* < 0.072; all *P* values ≥0.050) with measured white blood cell counts in both LBC1921 and LBC1936 (Additional file 1, Table S9). The incremental *R*^2^ upon the addition of the EpiScore to a linear regression model adjusting for age and sex was 3.4% (*P*=2.0x10^-8^) in LBC1936 and 4.5% (*P*=9.9x10^-6^) in LBC1921. The corresponding *R*^2^ for the polygenic score derived from a GWAS of 168,033 individuals in UK Biobank—a training sample approximately 18-times greater than the DNAm training sample—was 7.3% (*P*<2x10^-16^) and 6.9% (*P*=3.1x10^-8^), respectively. The additive incremental *R*^2^ from the two omics-based predictors was 10.7% in LBC1936 and 10.5% in LBC1921 (Fig. 1).

### Relationship between sample size and theoretical variance explained by a cognitive EpiScore

To see how the size of the training sample might affect the proportion of variance in *g* we could explain, we simulated several scenarios. Based on a training sample size of 10,000 and an (arbitrary) assumption of 100,000 CpGs affecting the trait, with a DNAm variance components estimate of 41.6%, we would expect a DNAm prediction *R*^2^ value of about 4.0% (following Formula 1 from [16]). This is very similar to the estimates obtained. If the training sample increased to 20,000 or 100,000 then the expected *R*^2^ should increase to around 8% and 29%, respectively.

### Comparison of the associations between measured cognitive ability and a cognitive EpiScore with health and lifestyle factors

We then tested how the EpiScore associated with health and lifestyle risk factors and how these compared to those observed for measured *g*. If the EpiScore yields similar effect sizes to measured *g* in its associations with these factors then it would strengthen its case for inclusion in prediction and risk stratification analyses for cognitive decline. In age- and sex-adjusted linear regression analyses with common risk factors of cognitive decline (smoking, years of education, BMI, lung function, walking speed, grip strength, high blood pressure, alcohol consumption, a depression questionnaire score, and an index of social deprivation), the EpiScore showed directionally consistent but weaker associations than measured *g* across both LBC1921 and LBC1936 (Fig. 1 and Additional file 1, Table S10)*.* The only exception was alcohol consumption, where the EpiScore outperformed measured *g*. A multiple regression model in LBC1936 with all covariates as predictors of measured *g* is presented in Additional file 1, Table S11. Upon addition of the EpiScore to the model, the incremental *R*^2^ estimate was 0.8% (*P*=1.8x10^-3^).

### Comparison of the associations between measured cognitive ability and a cognitive EpiScore with inflammatory protein levels

To see if the EpiScore and measured *g* metrics were comparable in terms of their associations with protein biomarkers, age- and sex-adjusted linear regression analyses were conducted with 70 Olink inflammatory protein levels in LBC1936. The EpiScore and measured *g* associations with the proteins were moderately concordant (*r* = 0.43). The EpiScore associated with 11 proteins (*P*<0.05/70) compared to 5 for measured *g* with two proteins, IL-6 and IL18.R1, overlapping both sets (Fig. 1 and Additional file 1, Table S12).

### Comparison of the associations between measured cognitive ability and a cognitive EpiScore with neurology protein levels and brain MRI measures

**Fig. 2:**
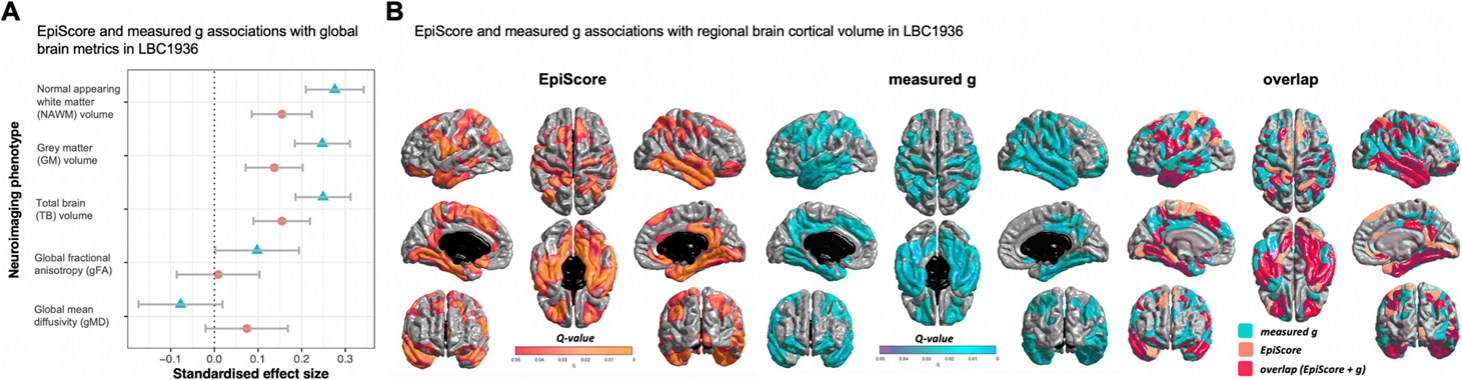
Measured and epigenetic cognitive ability associate with brain structure and show regional overlap with cortical loci. Cognitive ability measures with global brain imaging associations in LBC1936 with 95% confidence intervals; measured *g* (turquoise triangle), Epigenetic Score (EpiScore; orange circle) (**A**). Results of cortical volume at age 73 years regressed against cognitive *g* EpiScore (orange), measured *g* (turquoise) and the spatial extent of overlap (pink) in cortical loci. Colours, representing *q* values, are superimposed on an average surface template. A false discovery rate threshold of 0.05 is used to control for multiple comparisons; results are corrected for sex, age in days at brain scanning and intracranial volume (*n*=551) (**B**)

Finally, we compared the EpiScore and *g* associations with brain imaging outcomes and neurology protein levels. DNA methylation, structural brain MRI, and 90 Olink neurology-related proteins were also available at a follow-up wave of LBC1936 when participants were a mean age of 73 years (*n*=701 with proteins and *n*=551 with MRI). The EpiScore—protein associations mirrored those previously reported with measured fluid cognitive ability in the same dataset [17] with similar effect size estimates (*r* = 0.70). Thirteen EpiScore- and 15 measured *g*-protein associations were statistically significant (*P*<0.05/90) with 7 overlapping (Fig. 1 and Additional file 1, Table S12). There were associations with brain imaging measures of global volume (total brain, grey matter and normal appearing white matter volumes—Fig. 2 and Additional file 1, Table S13; Additional file 3). Furthermore, there were widespread associations between the EpiScore and regional brain cortical volume and thickness (Fig. 2 and Additional file 2, Figures S4-S5; Additional file 3), with significant overlap in cortical loci for both measured *g* and EpiScore. Overall, the EpiScore findings largely mirrored the associations between measured *g* and neuroimaging outcomes, albeit they were slightly smaller in magnitude.

## Discussion

This is the first variance component analysis of DNAm and cognitive function. We show substantial contributions to general cognitive function (*g*) in addition to the development of a novel epigenetic score with application to two independent test cohorts where it associated with measured cognitive ability.

We also show associations between the EpiScore and lifestyle factors and risk factors for dementia, circulating levels of neuroinflammatory proteins and brain MRI measures. As these findings reflected similar, albeit slightly weaker associations to those with measured *g*, a cognitive EpiScore may provide a useful tool to measure brain health in clinical settings and to aid in risk prediction of neurodegeneration.

Whereas many of the associations with measured cognitive ability in LBC1936 have been studied and discussed in detail previously (e.g., neurology proteins, brain MRI, health and lifestyle variables [17, 18]), we present for the first time the associations with inflammatory proteins. The EpiScore and measured *g* both associated with levels of IL-6 (a well-established correlate of cognitive ability [19]) and IL18.R1 is a subunit of the IL18 receptor that, with IL18, participates in neuroinflammatory and neurodegenerative processes [20]. The concordance of effect size estimates between the EpiScore and measured *g* variables with multiple outcomes highlights the utility of the former as a potential surrogate measure. The EpiScore-health findings mirrored associations between measured *g* and health across all assessed modalities. This included brain MRI, blood protein levels and general lifestyle outcomes. Higher EpiScores were linked to greater total brain, grey matter and white matter volumes, lower levels of neuroinflammatory proteins that have been linked to poorer brain health, and more positive lifestyle patterns (e.g. faster walking speed, greater grip strength, lower BMI and lower prevalence of hypertension).

We used a large homogeneous discovery cohort with consistent cognitive testing and DNA collection across all participants. This will minimise the biases that are an inherent problem in heterogeneous and small EWAS meta-analyses. The estimation of posterior effect sizes using BayesR+ has been shown to be robust to various potential sources of heterogeneity, including family structure [21] and population structure based on genetic or epigenetic principal components [6]. Nonetheless, replication of our findings in cohorts of different ages and backgrounds, and at different stages of neuro-development/degeneration will help to refine and generalise the estimates presented here. Whereas a near unit correlation was observed between the cognitive score residuals with and without a quadratic age term, modelling non-linear relationships may also aid locus discovery and improve external predictions.

Methylation-based predictors of cognitive function may improve longitudinal disease prediction and risk profiling of neurodegenerative health outcomes such as dementia. Here, the epigenetic variance components and prediction score were independent of genetic contributions; the EpiScore also reflected measured cognitive ability in its associations with a variety of biological and health-based traits. Unlike DNA differences which are largely fixed throughout life, DNAm differences may reflect environmental effects and phenotypic causation, directly through cognitive-related pathways or indirectly via related lifestyle and health outcomes. The EpiScore can therefore reflect consequences of processes linked to cognitive health. This is evident by the attenuation of the incremental *R*^2^ estimate from 3.4 to 0.8% in a multiple regression model that included age and sex plus 10 covariates that are established correlates/risk factors for cognitive ability and cognitive decline. The correlation with these risk factors and the independent contribution of the EpiScore from the polygenic score for prediction of measured *g* emphasises the environmental variance being captured by the EpiScore. Given the overwhelming contribution of CpGs with small effects to our estimates, increasing EWAS sample sizes will likely lead to both locus discovery and more accurate DNAm-based predictors of cognitive function. Future studies should investigate prospective associations of the EpiScore relative to measured *g*. Even if the former is capturing processes related to or downstream of cognitive function, it may still provide information for assessing risk of neurodegeneration. Whereas the associations of the current EpiScore with lifestyle and MRI variables are more modest than those observed for measured *g*, this gap is likely to narrow as the sample size of the training set increases. This has potential implications for studies of cognitive function across the lifespan where pen-and-paper testing is not possible or unreliable, such as during neurodevelopment or neurodegeneration. In the future, cognitive EpiScores may be help to monitor decline in brain health and to stratify individuals into risk groups years prior to a clinical diagnosis of dementia.

## Conclusions

As sample sizes increase, our ability to assess cognitive function from DNAm data may be informative in settings where cognitive testing is unreliable or unavailable.

## Methods

### The Generation Scotland Cohort

Details of the Generation Scotland: Scottish Family Health Study (GS) have been described in detail elsewhere [9, 22]. Briefly, GS comprises over 20,000 individuals comprehensively profiled for genetic, clinical, lifestyle and sociodemographic data. Around 8000 participants aged between 35 and 65 years were initially recruited through GP surgeries from five regions across Scotland. These participants were then asked to invite family members to join the study. Recruitment took place between 2006 and 2011 and the structure included relatives from up to three generations per family (around 5600 families participating in total). The age range of the cohort at the study baseline was 18 to 99 years. A subset of 9162 individuals from GS (aged 18 to 93 years, mean=49.7, SD=13.6) had genome-wide DNA methylation measured [23]. This subset was processed in two batches, hereafter referred to as “Set 1” and “Set 2”.

### Methylation preparation in Generation Scotland

Quality control was performed on Illumina HumanMethylationEPIC BeadChip DNA methylation data from blood samples of 5200 related individuals from Set 1, and 4583 genetically unrelated individuals from Set 2, also genetically unrelated to those in Set 1. Three Set 1 individuals who had answered “yes” to presence of all of 16 self-reported disease conditions in the study’s health questionnaire were excluded from the analysis. Filtering for outliers (*N*_Set1_=80; *N*_Set2_=83), sex mismatches (*N*_Set1_=19; *N*_Set2_=12), non-blood samples (*N*_Set1_=13), and poorly detected samples was performed (*N*_Set1_=18) [23]. Further filtering was then carried out to remove, non-autosomal and non-CpG sites (*N*=22,163), CpGs with missing values and poorly-detected CpGs (*N*_Set1_=5910; *N*_Set2_=8878). Five individuals with a self-reported diagnosis of Alzheimer’s disease were removed, along with those with missing covariate information or cognitive variables. Following filtering, 9162 complete cases remained comprising 4901 Set 1 individuals and 4261 Set 2 individuals, with 764,525 CpGs in common between the two.

### Cognitive Phenotypes in Generation Scotland

Six cognitive phenotypes were assessed in this study: logical memory, digit symbol test score, verbal fluency, vocabulary, general cognitive ability, and general fluid cognitive ability. The logical memory phenotype (verbal declarative memory) was calculated from the Wechsler Logical Memory test, taking the sum of immediate and delayed recall of one oral story [24]. The digit symbol phenotype is often used as a measure of processing speed and was calculated from the Wechsler Digit Symbol Substitution test in which participants must recode digits to symbols over a 120 second period [25]. The verbal fluency phenotype is often used as a measure of executive functioning and was derived from the phonemic verbal fluency test, using the letters C, F and L, each for 1 min [26]. Vocabulary was measured using the Mill Hill Vocabulary Scale, junior and senior synonyms combined [27]. Outliers were defined as scores >3.5 standard deviations above or below the mean and were removed prior to analysis. General fluid cognitive ability (*g*_*f*_) was calculated from the first unrotated principal component of logical memory, verbal fluency and digit symbol tests. General cognitive ability (*g*) was derived from the first unrotated principal component from the same variables plus vocabulary.

### The Lothian Birth Cohorts of 1921 and 1936

The mean posterior effect sizes for the EWAS model of general cognitive function, *g*, were used to generate an epigenetic predictor in two independent datasets, the Lothian Birth Cohort 1936 (LBC1936) and the Lothian Birth Cohort 1921 (LBC1921).

The LBC are studies of cognitive ageing in older adults from the area around Edinburgh, Scotland [28, 29]. Briefly, participants were born in either 1921 or 1936 and completed the Scottish Mental Survey of 1932 or 1947 at age 11. From age 70 (LBC1936) and age 79 (LBC1921), they were assessed triennially for a variety of health and lifestyle outcomes, with DNA collected at each visit.

### Methylation preparation in the Lothian Birth Cohorts

We considered blood-based DNA methylation data from the age 70 (LBC1936) and age 79 (LBC1921) samples. Methylation was assessed on the Illumina 450k array—the predecessor of the EPIC array. Processing and quality control have been described previously [1, 30, 31]. This included steps to remove methylation samples and individuals with poor quality control measures, along with individuals who had mismatching genotypes or predicted sex information. DNA methylation was measured at three time points (set 1, set 2 and set 3) and comprised 2,195, 996 and 552 samples, respectively. Prior to quality control, each set had 485,512 CpGs. Twenty-three duplicate samples were removed from set 2. Set 1 and set 2 had 123 duplicates between them, and a sample was removed from each duplicate pair (108 from set 1, 15 from set 2). Sets 1 and 2 were then combined (set12). Ten duplicates were removed from set 3. There were also 31 duplicates between set 3 and set 12. 26 samples were removed from set 3 and five were removed from set12. The three sets were combined (set123) and comprised 3556 samples. Samples and CpGs were filtered on low call rates (CpGs with a detection *p* value greater than 0.01), with a threshold of 95% for both samples and CpGs. 3525 samples and 470,278 CpGs remained after this step. Finally, sex chromosome CpGs were removed, leaving a dataset comprising 459,309 CpGs and 3525 samples. The current study used a subset from this dataset comprising 381,846 CpGs (overlapping with those included in the Generation Scotland analyses) for 861 LBC1936 (436 LBC1921) individuals—34 LBC1936 individuals were excluded due to DNAm being assessed as part of a separate analysis batch.

### Cognitive Epigenetic Score (EpiScore) in the Lothian Birth Cohorts of 1921 and 1936

Within each LBC study, each CpG was scaled to mean 0, variance 1 with missing values mean imputed (i.e. set to 0) prior to multiplication by the mean CpG weights (for all available CpGs) and summation to give the epigenetic score.

### Cognitive Polygenic Score in the Lothian Birth Cohorts

A polygenic score for cognitive ability was derived from *Z* scores from all possible SNPs (GWAS *P*≤1) in a UK Biobank GWAS of verbal numerical reasoning (*n*=168,033) [32] and applied to LBC1936 and LBC1921 genotype data using default settings in the PRSice software [33, 34]. The *P*≤1 threshold for polygenic scores of cognitive ability has similar predictive performance as scores built using more conservative thresholds [35].

### Cognitive Phenotypes in the Lothian Birth Cohorts

In LBC1936 at Wave 1 (mean age 70 years), general cognitive ability, *g*, was defined as the first unrotated principal component (that accounted for 52% of the variance) from a PCA of six cognitive tests from the Wechsler Adult Intelligence Scale-III UK (matrix reasoning, letter number sequencing, block design, symbol search, digit symbol and digit span backward) [25] plus a test of vocabulary (National Adult Reading Test) [36].

A similar approach was taken in LBC1921 at Wave 1 (mean age 79 years), where we considered the first unrotated principal component (that also accounted for 51% of the variance) from a PCA of four cognitive tests: Raven’s Standard Progressive Matrices [27], letter-number sequencing [25], digit symbol coding [25], and the National Adult Reading Test [36]). The cognitive tests were completed at the same visit that the blood was drawn for DNA profiling in both LBC studies.

### Statistical analysis

#### Variance components analysis and EWAS using BayesR+

BayesR+ was used for the EWAS and to estimate the proportion of variance in cognitive traits explained by genetics and DNA methylation. BayesR+ is a software implemented in C++ for performing Bayesian penalised regression and Gaussian mixture-based variance partitioning on complex traits [6]. The joint and conditional effects of methylation sites (*n*=764,525) on cognitive traits were examined. Phenotypic and methylation data were scaled to mean zero and unit variance. The prior distribution comprised a series of Gaussian distributions that corresponded to effect sizes of different magnitudes (i.e. methylation sites with small, medium and large effect sizes), as well as a discrete spike at zero which allows for the omission of probes with non-identifiable effects. The prior mixture variances were set to 0.0001, 0.001 and 0.01. Phenotypes were corrected for age, sex, BMI and epigenetic smoking score [10], and the DNA methylation data were corrected for batch, age, sex and epigenetic smoking. Adding a quadratic term for age in the correction of cognitive phenotypes made negligible differences to the trait residuals (*r*>0.98 between linear model residuals for each trait).

#### Estimating the variance components and individual CpG effects

To obtain estimates of variance accounted for in cognitive traits by methylation data and individual CpG associations with the cognitive test scores, Gibbs sampling was performed to sample over the posterior distribution conditional on the input data. The Gibbs algorithm consisted of 10,000 samples and 5000 samples of burn-in after which a thinning of 5 samples was applied to reduce autocorrelation. The process was repeated over four chains, initializing a different random number seed for each chain. The last 250 iterations from each chain were combined for downstream analyses. For the EWAS, CpGs within 2.5kb and highly correlated (absolute Pearson correlation >0.5) with a lead CpG with posterior inclusion probability greater than 0.2 were grouped together. For each probe group, we calculated the proportion of iterations for which at least one probe was included in the model, yielding the group posterior inclusion probability. We then calculated the average (across the 1000 iterations) sum of the squared regression coefficients for the probe group to give the contribution of the group to the total variance. Finally, we highlighted the lead CpG for the groups where the combined posterior inclusion probability was >0.80. The variance components estimates are taken as the mean sum of squared standardised mean posterior effect sizes across the 1000 iterations with the 2.5%ile and 97.5%ile forming the 95% credible interval.

#### Sensitivity analyses

Sensitivity analyses were performed using OSCA to estimate the proportion of variance explained in cognitive tests by epigenetic data. OSCA is a software tool designed for the analysis of complex traits using multiple omics data types, including genome-wide DNAm data. Omics relationship matrices (ORMs) were estimated using all probes to determine inter-individual relationships, analogous to a genomic relationship matrix (GRM), which is used to estimate SNP-based heritability. ORMs were computed separately using DNAm data from Generation Scotland Set 2 (unrelated individuals) and Sets 1 and 2 combined. Each ORM was subsequently fitted to a mixed linear model to estimate the variance explained by all DNAm probes using the restricted maximum likelihood (REML) method. These analyses were undertaken to confirm that the results obtained were not a function of the analysis method used.

#### GWAS and combined GWAS/EWAS analyses using BayesR+

Genetic effects at 560,797 SNPs (minor allele frequency > 1%; scaled to mean 0, variance 1) from the Illumina HumanOmniExpressExome-8 v1.0 Bead Chip or Illumina HumanOmniExpressExome-8 v1.2 Bead Chip were examined [37], setting prior mixture variances to 0.00001, 0.0001 and 0.001. For the genetic analysis, phenotypes were pre-corrected for age, sex and 20 genetic PCs. To estimate the additive and independent effects of DNAm and genetic data on complex traits, a combined analysis was run—using the phenotype corrections specified for the EWAS model—setting prior mixture variances as above.

#### EpiScore associations with measured *g* in the Lothian Birth Cohorts

There were 844 LBC1936 (427 LBC1921) individuals with cognitive, epigenetic and polygenic score data. Linear regression was used to test for an association between the predicted epigenetic score (predictor) and measured general cognitive ability (outcome), in models adjusting for age, sex and the polygenic score.

#### EpiScore and measured *g* associations with common risk factors in the Lothian Birth Cohorts

Age- and sex-adjusted linear regression models were used to relate the Epigenetic Score (EpiScore) for *g* and the measured *g* score (predictors) with common risk factors (outcomes) for cognitive decline, frailty and dementia. Each outcome was modelled independently in a separate regression model. The outcomes studied were body mass index (BMI in kg/m^2^); years of education; self-reported smoking (ever versus never); self-reported weekly units of alcohol; self-reported high blood pressure (yes/no); lung function (forced expiratory volume in one second) adjusted for age, sex, and height; time taken to walk 6 m (seconds); socioeconomic deprivation (Scottish Index of Multiple Deprivation in LBC1936 and social grades based on highest reached occupation [38] in LBC1921); and depression (HADS-D total from the Hospital Anxiety and Depression questionnaire) [28, 39, 40]. Prior to the analyses, BMI, 6-m walk time and units of alcohol were log transformed to reduce skew—a constant of one was added to the alcohol units before the log transformation.

#### EpiScore and measured *g* associations with neuroinflammatory proteins levels in the Lothian Birth Cohort 1936

An Olink panel of inflammation-related proteins [41], measured on blood samples at age 70 years in LBC1936, were related to both EpiScore *g* and measured *g* in age- and sex-adjusted linear regression models. An additional panel of Olink neurology-related proteins [17], measured on blood samples at age 73 years in LBC1936, were related to EpiScore *g*, which was derived from DNAm assessed from the same sample (analysis *n*=701). Quality control of the DNAm was identical to the age 70 samples. Processing occurred in two sets (*N*_Set1_=256, *N*_Set2_=445) where CpG sites were independently scaled to mean zero and variance one, prior to combining into a single variable. Each of the 70 inflammatory and 90 neurology proteins were adjusted via rank-based inverse normal transformations and regressed on age, sex and four genetic ancestry components as previously described [42]. Linear regression model assumptions were visually inspected via regression diagnostic plots.

#### EpiScore and measured *g* associations with brain MRI variables in the Lothian Birth Cohort 1936

Structural and diffusion tensor (DTI) MRI acquisition and processing in LBC1936 were performed at Wave 2 (age 73 years) according to an open-access protocol [43]. Total brain, grey matter and normal-appearing white matter (NAWM) volumes were calculated using a semi-automated multi-spectral fusion method [44]. Intracranial volume was determined semi-automatically using Analyze 11.0^TM^. White matter microstructural parameters fractional anisotropy (FA) and mean diffusivity (MD) were derived for 12 major tracts of interest: corpus callosum genu and splenium, bilateral frontal cingulum, arcuate, uncinate and superior longitudinal fasciculi and bilateral anterior thalamic radiation. These were obtained using probabilistic neighbourhood tractography in TractoR (https://www.tractor-mri.org.uk) [45, 46] as applied to BEDPOSTX/ProbTrackX in FSL (https://fsl.fmrib.ox.ac.uk) [47]. Participants were excluded if they had self-reported history of dementia or signs of cognitive impairment (Mini Mental State Examination score < 24/30); after exclusions, a total of 590 participants had complete cognitive, epigenetic and global neuroimaging data, and of these, 551 participants had complete and vertex-wise neuroimaging data. Localised associations between cognitive measures and vertex-wise cortical volume and thickness were performed using linear regression, controlling for age, sex and intracranial volume. The SurfStat MATLAB toolbox (http://www.math.mcgill.ca/keith/surfstat) for Matrix Laboratory R2012a (The MathWorks, Inc., Natick, MA, USA) was used to carry out analyses. Statistical maps of association magnitude and valence (*t*-maps) and significance (*q* maps; *p* values corrected for multiple comparisons using a false discovery rate with a *q* value of 0.05 across all 327,684 vertices on the cortical surface) were presented.

## Supplementary Information

**Additional file 1: Supplementary Tables 1-13**. **Table S1:** Generation Scotland cohort summary. **Table S2:** Mean variance accounted for by the effects of DNA methylation (DNAm) and genome-wide DNA single nucleotide polymorphisms (GWAS) alone, DNAm data conditioned on GWAS data (DNAmAdjGWAS), and the additive effects of DNAm and GWAS data for six cognitive traits. **Table S3:** Comparison of epigenetic variance components estimates between BayesR+ and a linear mixed model approach, OSCA. **Table S4:** Contribution of mixtures with small, medium, and large effect sizes (variances of 0.01%, 0.1%, and 1%, respectively) to the mean variance accounted for by the effects of DNA methylation (DNAm) for the cognitive traits. **Table S5:** List of blood-based CpG sites that are with strong evidence (Group PIP>0.95) for association with general cognitive function in Generation Scotland. PIP: Posterior Inclusion Probability. **Table S6:** Epigenome-wide association study (EWAS) catalog lookup of three CpGs with strong (Group PIP>0.95) associations with general cognitive ability. **Table S7:** Lookup of CpGs associated with cognitive abilities and Alzheimer’s disease. **Table S8:** LBC1921 and LBC1936 Cohort Summaries. **Table S9:** Correlations between measured white blood cell counts and cognitive EpiScore in LBC1921 and LBC1936. **Table S10:** Age- and sex-adjusted linear regression associations between the cognitive *g* Epigenetic Score (EpiScore) and measured *g* score and traits associated with cognitive ageing and dementia (outcomes) in the Lothian Birth Cohorts 1936 and 1921. **Table S11:** Multiple regression output from a saturated model comprised of measured *g* (outcome) age, sex, 10 health and lifestyle traits, and the EpiScore for general cognitive function (predictors). **Table S12:** Age- and sex-adjusted linear regression associations between 70 inflammation-related and 90 neurology related proteins (outcomes) with the cognitive *g* Epigenetic Score (EpiScore) and measured cognitive ability in the Lothian Birth Cohort 1936. **Table S13:** Age at MRI scan- and sex-adjusted linear regression associations between the Epigenetic *g* Score (EpiScore) and measured *g* score and traits associated with global neuroimaging outcomes in the Lothian Birth Cohorts 1936.

**Additional file 2: Supplementary Figures 1-5**. **Figure S1:** Plot of the epigenetic smoking variable (EpiSmoker) against self-reported smoking status (current, former, never) in Generation Scotland (*n*=9,162). **Figure S2:** Median effects observed for DNA methylation probes with posterior inclusion probabilities (PIPs) > 0.8 across four cognitive tests and two composite measures (no probes identified for logical memory). **Figure S3:** Scatter Plot of Epigenetic *g* Score by Measured *g* Score in the Lothian Birth Cohort 1936 (LBC1936) and the Lothian Birth Cohort 1921 (LBC1921). **Figure S4:** Regional cortical volume regressed against measured *g* (**left**) and EpiScore *g* (**middle**), colours denote the magnitude (T-maps; top, **A-B**) and significance (Q values; bottom, **D-E**) of the negative associations between cognitive measures and brain cortical volume. Panel (**C**) shows the percentage attenuation for the significant associations between EpiScore and cortical volume when also controlling for measured *g*. (**F**) shows the spatial extent overlap (green) in cortical loci that exhibit FDR-corrected unique associations. **Figure S5:** Regional cortical thickness regressed against measured *g* (**left**) and EpiScore *g* (**middle**), colours denote the magnitude (T-maps; top, **A-B**) and significance (Q values; bottom, **D-E**) of the negative associations between cognitive measures and brain cortical thickness. Panel (**C)** shows the percentage attenuation for the significant associations between EpiScore and cortical thickness when also controlling for measured *g*. (**F**) shows the spatial extent overlap (green) in cortical loci that exhibit FDR-corrected unique associations.

**Additional file 3.** Online Imaging Methods.

**Additional file 4.** Review history.

## Data Availability

According to the terms of consent for GS participants, access to data must be reviewed by the GS Access Committee. Applications should be made to access@generationscotland.org. Lothian Birth Cohort data are available on request from the Lothian Birth Cohort Study, University of Edinburgh (simon.cox@ed.ac.uk). Lothian Birth Cohort data are not publicly available due to them containing information that could compromise participant consent and confidentiality. All code is available with open access at the following GitHub repository https://github.com/marioni-group/ewas_of_cognitive_function (Zenodo 10.5281/zenodo.5794175 [48]).
