# Supplementary material for: Blood-based epigenome-wide analyses of cognitive abilities
Source: Genome Biol. 2022 Jan 17;23:26. doi: 10.1186/s13059-021-02596-5 (PMC8762878; doi:10.1186/s13059-021-02596-5)
Supplement: Supplementary file 1 — Additional file 1: Supplementary Tables 1-13. Table S1: Generation Scotland cohort summary. Table S2: Mean variance accounted for by the effects of DNA methylation (DNAm) and genome-wide DNA single nucleotide polymorphisms (GWAS) alone, DNAm data conditioned on GWAS data (DNAmAdjGWAS), and the additive effects of DNAm and GWAS data for six cognitive traits. Table S3: Comparison of epigenetic variance components estimates between BayesR+ and a linear mixed model approach, OSCA. Table S4: Contribution of mixtures with small, medium, and large effect sizes (variances of 0.01%, 0.1%, and 1%, respectively) to the mean variance accounted for by the effects of DNA methylation (DNAm) for the cognitive traits. Table S5: List of blood-based CpG sites that are with strong evidence (Group PIP>0.95) for association with general cognitive function in Generation Scotland. PIP: Posterior Inclusion Probability. Table S6: Epigenome-wide association study (EWAS) catalog lookup of three CpGs with strong (Group PIP>0.95) associations with general cognitive ability. Table S7: Lookup of CpGs associated with cognitive abilities and Alzheimer’s disease. Table S8: LBC1921 and LBC1936 Cohort Summaries. Table S9: Correlations between measured white blood cell counts and cognitive EpiScore in LBC1921 and LBC1936. Table S10: Age- and sex-adjusted linear regression associations between the cognitive g Epigenetic Score (EpiScore) and measured g score and traits associated with cognitive ageing and dementia (outcomes) in the Lothian Birth Cohorts 1936 and 1921. Table S11: Multiple regression output from a saturated model comprised of measured g (outcome) age, sex, 10 health and lifestyle traits, and the EpiScore for general cognitive function (predictors). Table S12: Age- and sex-adjusted linear regression associations between 70 inflammation-related and 90 neurology related proteins (outcomes) with the cognitive g Epigenetic Score (EpiScore) and measured cognitive ability in the Lothian Birth Coho [file 13059_2021_2596_MOESM1_ESM.docx]

**Table S1:** Generation Scotland cohort summary

|  | N | Mean | SD |
| --- | --- | --- | --- |
| Age (years) | 9162 | 49.8 | 13.6 |
| Digit Symbol | 9162 | 71.2 | 16.5 |
| Verbal Fluency | 9162 | 40.0 | 11.5 |
| Vocabulary | 9162 | 30.5 | 4.5 |
| Logical Memory | 9162 | 31.1 | 7.9 |
| Body Mass Index (kg/m^2^) | 9162 | 26.9 | 5.2 |
| EpiSmoker^*^ | 9162 | 2.09 | 4.5 |
|  | N | % |  |
| Sex |  |  |  |
| Male | 3752 | 41.0 |  |
| Female | 5410 | 59.0 |  |

^*^ A plot of the epigenetic smoker (EpiSmoker) variable against self-reported smoking (current, former, never) is provided in **Supplementary Figure 1**.

**Table S2:** Mean variance accounted for by the effects of DNA methylation (DNAm) and genome-wide DNA single nucleotide polymorphisms (GWAS) alone, DNAm data conditioned on GWAS data (DNAmAdjGWAS), and the additive effects of DNAm and GWAS data for six cognitive traits. General fluid cognitive ability (*g_f_*) was calculated from the first unrotated principal component of logical memory, verbal fluency, and digit symbol tests. General cognitive ability (*g*) was derived from the first unrotated principal component from the same variables plus vocabulary

|  | **DNAm** | | | **GWAS** | | | **DNAmAdjGWAS** | | | **DNAm+GWAS** | | |
| --- | --- | --- | --- | --- | --- | --- | --- | --- | --- | --- | --- | --- |
|  | **Mean** | **2.5%ile** | **97.5%ile** | **Mean** | **2.5%ile** | **97.5%ile** | **Mean** | **2.5%ile** | **97.5%ile** | **Mean** | **2.5%ile** | **97.5%ile** |
| Digit Symbol | 0.242 | 0.125 | 0.385 | 0.134 | 0.000 | 0.396 | 0.220 | 0.115 | 0.338 | 0.330 | 0.145 | 0.533 |
| Logical Memory | 0.053 | 0.000 | 0.216 | 0.081 | 0.000 | 0.344 | 0.045 | 0.000 | 0.196 | 0.124 | 0.000 | 0.419 |
| Verbal Fluency | 0.230 | 0.121 | 0.378 | 0.227 | 0.000 | 0.532 | 0.192 | 0.078 | 0.390 | 0.461 | 0.198 | 0.648 |
| Vocabulary | 0.458 | 0.373 | 0.541 | 0.429 | 0.244 | 0.601 | 0.415 | 0.294 | 0.533 | 0.704 | 0.599 | 0.796 |
| g_f_ | 0.292 | 0.173 | 0.420 | 0.243 | 0.000 | 0.466 | 0.253 | 0.151 | 0.373 | 0.418 | 0.200 | 0.626 |
| g | 0.416 | 0.310 | 0.530 | 0.379 | 0.183 | 0.529 | 0.350 | 0.248 | 0.467 | 0.638 | 0.500 | 0.735 |
|  |  |  |  |  |  |  |  |  |  |  |  |  |

**Table S3:** Comparison of epigenetic variance components estimates between BayesR+ and a linear mixed model approach, OSCA. OSCA^1^ analyses included an epigenetic relationship matrix on all 9,162 participants; OSCA^2^ analyses included an epigenetic relationship matrix on a genetically unrelated subset of 4,261 participants who were processed in a single methylation batch (“Set 2” – **Online Methods**).

|  | **DNAm variance component estimate** | | |
| --- | --- | --- | --- |
|  | **BayesR+ (mean)** | **OSCA^1^**  **(mean, SE)** | **OSCA^2^**  **(mean, SE)** |
| Logical Memory | 0.053 | 0.062 (0.03) | 0.142 (0.08) |
| Digit Symbol | 0.242 | 0.257 (0.04) | 0.231 (0.07) |
| Verbal Fluency | 0.230 | 0.254 (0.04) | 0.168 (0.07) |
| Vocabulary | 0.458 | 0.484 (0.03) | 0.604 (0.06) |
| g_f_ | 0.292 | 0.313 (0.04) | 0.417 (0.08) |
| g | 0.416 | 0.434 (0.03) | 0.584 (0.07) |

**Table S4:** Contribution of mixtures with small, medium, and large effect sizes (variances of 0.01%, 0.1%, and 1%, respectively) to the mean variance accounted for by the effects of DNA methylation (DNAm) for the cognitive traits.

|  | **DNAm effect sizes** | | | |
| --- | --- | --- | --- | --- |
|  | **Small** | **Medium** | **Large** | **Total** |
| Logical Memory | 0.025 | 0.014 | 0.014 | 0.053 |
| Digit Symbol | 0.138 | 0.079 | 0.025 | 0.242 |
| Verbal Fluency | 0.101 | 0.114 | 0.016 | 0.231 |
| Vocabulary | 0.330 | 0.112 | 0.016 | 0.458 |
| *g*_f_ | 0.196 | 0.073 | 0.023 | 0.292 |
| *g* | 0.313 | 0.073 | 0.030 | 0.416 |
|  |  |  |  |  |

**Table S5:** List of blood-based CpG sites that are with strong evidence (Group PIP>0.95) for association with general cognitive function in Generation Scotland. PIP: Posterior Inclusion Probability.

For the EWAS, CpGs within 2.5kb and highly correlated (absolute Pearson correlation >0.5) with a lead CpG with posterior inclusion probability greater than 0.2 were grouped together. For each probe group (with number of CpGs – **nCpG**), we calculated the proportion of iterations for which at least one probe was included in the model, yielding the group posterior inclusion probability (**Group PIP**). We then calculated the sum of the squared regression coefficients at each iteration for the probe group to give the contribution of the group to the total variance (**Group R^2^**). Finally, we highlighted the lead CpG for the groups.

| **Lead CpG** | **Group PIP** | **Group R^2^** | **Nearest Gene** | **nCpG** | **Lead PIP** | **Lead Median Beta** | **Lead Beta2.5** | **Lead Beta97.5** |
| --- | --- | --- | --- | --- | --- | --- | --- | --- |
| cg09984392 | 1.013 | 0.005 | *SQLE* | 4 | 0.965 | 0.072 | 0.000 | 0.104 |
| cg13108341 | 1.000 | 0.009 | *DNAH9* | 1 | 1.000 | -0.092 | -0.124 | -0.054 |
| cg05325763 | 0.952 | 0.005 | *CPT1A* | 5 | 0.870 | -0.070 | -0.108 | 0.000 |

**Table S6:** Epigenome-wide association study (EWAS) catalog lookup of three CpGs with strong (Group PIP>0.95) associations with general cognitive ability. Lookup is restricted to associations at P<3.6x10^-8^. * denotes multiple models in study, where summary statistics are presented for the model with the most significant association.

| **CpG** | **First Author** | | **Study ID** | | **EWAS Trait** | | | **Beta** | **SE** | **P** |  |  |  |  |
| --- | --- | --- | --- | --- | --- | --- | --- | --- | --- | --- | --- | --- | --- | --- |
| cg13108341 | Bermingham M | | 30935889 | | Forced Vital Capacity | | | -0.032 | NA | 2.6x10^-8^ |  |  |  |  |
| cg09984392 | Mulder RH | | 33450751 | | Age^*^ | | | 0.0031 | 0.00011 | 0 |  |  |  |  |
| cg09984392 | Battram T | | 30602389 | | Tissue | | | -0.0694 | 0.005 | 1.2x10^-28^ |  |  |  |  |
| cg09984392 | Petersen A-K | | 24014485 | | 1-linoleoylglycerophosphoethanolamine^*^ | | | 4.26 | 0.11 | 6.50x10^-11^ |  |  |  |  |
| cg09984392 | Singmann P | | 26500701 | | Sex | | | NA | NA | 1.52x10^-8^ |  |  |  |  |
|  |  |  | |  | |  |  | | | | |  |  |  |

**Table S7:** Lookup of CpGs associated with cognitive abilities and Alzheimer’s disease. PIP (Posterior Inclusion Probability) is presented for *g*, as reported in the current study.

| **CpG** | **PIP** | **Study** |
| --- | --- | --- |
| cg13344806 | 0.012 | Lunnon et al. |
| cg05667256 | 0.017 | Lunnon et al. |
| cg03048889 | 0.012 | Lunnon et al. |
| cg17074958 | 0.016 | Lunnon et al. |
| cg05242218 | 0.006 | Lunnon et al. |
| cg14067233 | 0.014 | Lunnon et al. |
| cg07401045 | 0.011 | Lunnon et al. |
| cg01986016 | 0.011 | Roubroeks et al. |
| cg10906729 | 0.017 | Roubroeks et al. |
| cg05888755 | 0.017 | Roubroeks et al. |
| cg00072689 | 0.011 | Roubroeks et al. |
| cg20471691 | 0.008 | Roubroeks et al. |
| cg15435170 | 0.008 | Roubroeks et al. |
| cg18684142 | 0.008 | Roubroeks et al. |
| cg17179862 | 0.009 | Roubroeks et al. |
| cg19828220 | 0.011 | Roubroeks et al. |
| cg03803541 | 0.006 | Roubroeks et al. |
| cg16848873 | 0.008 | Roubroeks et al. |
| cg18181703 | 0.033 | Marioni et al. |
| cg04513006 | 0.024 | Marioni et al. |
| cg03366574 | 0.011 | Marioni et al. |
| cg16201957 | 0.016 | Marioni et al. |
| cg21450381 | 0.009 | Marioni et al. |
| cg12507869 | 0.012 | Marioni et al. |

**Table S8.** LBC1921 and LBC1936 Cohort Summaries

|  | **LBC1921** | | |  |  | **LBC1936** | | |  |
| --- | --- | --- | --- | --- | --- | --- | --- | --- | --- |
|  | N | Mean | SD |  |  | N | Mean | SD |  |
| Age (years) | 427 | 79.13 | 0.58 |  |  | 844 | 69.59 | 0.83 |  |
| BMI (kg/m^2^) | 424 | 26.19 | 4.05 |  |  | 843 | 27.78 | 4.34 |  |
| Alcohol (units per week) | 427 | 5.47 | 8.99 |  |  | 844 | 10.19 | 13.71 |  |
| Deprivation* | 426 | 2.22 | 0.88 |  |  | 838 | 4652 | 1882 |  |
| 6m Walk (seconds) | 423 | 4.71 | 1.92 |  |  | 840 | 3.84 | 1.21 |  |
| Education (years) | 425 | 11.00 | 2.52 |  |  | 844 | 10.74 | 1.11 |  |
| Depression (HADS) | 425 | 3.52 | 2.29 |  |  | 842 | 2.73 | 2.18 |  |
| Grip strength (kg) | 424 | 26.2 | 8.91 |  |  | 840 | 29.55 | 10.15 |  |
| Lung function (FEV_1_; litres) | 424 | 1.88 | 0.62 |  |  | 842 | 2.36 | 0.67 |  |
|  | N | % |  |  |  | N | % |  |  |
| Sex |  |  |  |  |  |  |  |  |  |
| Male | 169 | 39.6 |  |  |  | 426 | 50.47 |  |  |
| Female | 258 | 60.4 |  |  |  | 418 | 49.53 |  |  |
| Hypertension |  |  |  |  |  |  |  |  |  |
| Yes | 171 | 40.5 |  |  |  | 348 | 41.23 |  |  |
| No | 251 | 59.5 |  |  |  | 496 | 58.77 |  |  |
| Ever smoked |  |  |  |  |  |  |  |  |  |
| Current | 29 | 6.81 |  |  |  | 95 | 11.26 |  |  |
| Former | 192 | 45.07 |  |  |  | 355 | 42.06 |  |  |
| Never | 205 | 48.12 |  |  |  | 394 | 46.68 |  |  |

Deprivation was measured using social grades based on highest reached occupation in LBC1921, and the Scottish Index of Multiple Deprivation (SIMD) in LBC1936.

**Table S9.** Correlations between measured white blood cell counts and cognitive EpiScore in LBC1921 and LBC1936.

| **Cell** | **Cor** | **P** | **Study** |
| --- | --- | --- | --- |
| Neutrophils | -0.016 | 0.65 | LBC1936 |
| Lymphocytes | -0.039 | 0.26 | LBC1936 |
| Monocytes | 0.0027 | 0.94 | LBC1936 |
| Eosinophils | 0.017 | 0.61 | LBC1936 |
| Basophils | -0.068 | 0.05 | LBC1936 |
| Neutrophils | 0.072 | 0.15 | LBC1921 |
| Lymphocytes | -0.0029 | 0.95 | LBC1921 |
| Monocytes | -0.063 | 0.2 | LBC1921 |
| Eosinophils | -0.062 | 0.21 | LBC1921 |
| Basophils | 0.044 | 0.37 | LBC1921 |

**Table S10.** Age- and sex-adjusted linear regression associations between the cognitive *g* Epigenetic Score (EpiScore) and measured *g* score and traits associated with cognitive ageing and dementia (outcomes) in the Lothian Birth Cohorts 1936 and 1921. All continuous variables are standardised to mean 0 and variance 1.A significance threshold was set at P<0.05 (Bonferroni P = 0.05/40=1.25x10^-3^). Nominally significant associations (P < 0.05) are presented in bold.

|  | **LBC1936** | | | | | | **LBC1921** | | | | | |
| --- | --- | --- | --- | --- | --- | --- | --- | --- | --- | --- | --- | --- |
|  | **EpiScore** | | | **Measured g** | | | **EpiScore** | | | **Measured g** | | |
| **Trait** | **Effect** | **SE** | **P** | **Effect** | **SE** | **P** | **Effect** | **SE** | **P** | **Effect** | **SE** | **P** |
| BMI | -0.095 | 0.035 | **6.10E-03** | -0.153 | 0.0353 | **1.58E-05** | -0.07 | 0.049 | 0.155 | -0.074 | 0.05 | 0.139 |
| Alcohol | 0.089 | 0.033 | **7.40E-03** | 0.0629 | 0.0342 | 0.0662 | 0.111 | 0.047 | **0.0177** | 0.084 | 0.047 | 0.0737 |
| Depression | -0.122 | 0.035 | **4.40E-04** | -0.198 | 0.0352 | **2.36E-08** | -0.058 | 0.049 | 0.236 | -0.087 | 0.049 | 0.0763 |
| Deprivation | 0.133 | 0.034 | **9.30E-05** | 0.342 | 0.0331 | **1.36E-23** | -0.144 | 0.048 | **2.80E-03** | -0.434 | 0.044 | **4.39E-21** |
| 6m Walk | -0.09 | 0.034 | **7.90E-03** | -0.246 | 0.0342 | **1.37E-12** | -0.116 | 0.048 | **0.0167** | -0.167 | 0.049 | **6.38E-04** |
| Education | 0.128 | 0.034 | **1.90E-04** | 0.455 | 0.0319 | **1.99E-41** | 0.098 | 0.048 | **0.0424** | 0.471 | 0.042 | **2.58E-25** |
| Grip strength | 0.072 | 0.021 | **8.00E-04** | 0.142 | 0.0215 | **6.51E-11** | 0.109 | 0.032 | **7.30E-04** | 0.145 | 0.032 | **8.61E-06** |
| Lung function | 0.067 | 0.035 | 0.055 | 0.176 | 0.0353 | **7.80E-07** | 0.102 | 0.049 | **0.0366** | 0.192 | 0.049 | **9.42E-05** |
| Ever smoked | -0.033 | 0.034 | 0.34 | -0.091 | 0.035 | **0.01** | -0.11 | 0.048 | **0.0206** | -0.016 | 0.050 | 0.740 |
| Hypertension | -0.032 | 0.035 | 0.35 | -0.098 | 0.036 | **0.006** | -0.003 | 0.049 | 0.943 | 0.027 | 0.049 | 0.583 |

**Table S11.** Multiple regression output from a saturated model comprised of measured g (outcome) age, sex, 10 health and lifestyle traits, and the EpiScore for general cognitive function (predictors).

| **Predictor** | **Beta** | **SE** | **P** |
| --- | --- | --- | --- |
| (Intercept) | 0.166 | 0.069 | 0.016 |
| Age | -0.172 | 0.030 | 9.65E-09 |
| Sex | -0.265 | 0.096 | 5.76E-03 |
| **EpiScore** | **0.093** | **0.030** | **1.81E-03** |
| Education | 0.323 | 0.031 | 6.24E-24 |
| BMI | -0.033 | 0.031 | 0.276 |
| Smoking | 0.012 | 0.061 | 0.848 |
| Alcohol | 0.012 | 0.031 | 0.707 |
| Hypertension | -0.086 | 0.059 | 0.148 |
| Depression | -0.077 | 0.030 | 0.011 |
| Deprivation | 0.177 | 0.032 | 4.20E-08 |
| 6m Walk | -0.067 | 0.033 | 0.046 |
| Grip strength | 0.142 | 0.050 | 4.39E-03 |
| Lung function | 0.032 | 0.031 | 0.309 |

**Table S12.** Age- and sex-adjusted linear regression associations between 70 inflammation-related and 90 neurology related proteins (outcomes) with the cognitive *g* Epigenetic Score (EpiScore) and measured cognitive ability in the Lothian Birth Cohort 1936. Neurology associations with measured fluid cognitive ability are reported in [Supplementary Data 2](https://static-content.springer.com/esm/art%3A10.1038%2Fs41467-019-14161-7/MediaObjects/41467_2019_14161_MOESM4_ESM.xlsx) of Harris et al. Nature Communications.

| **Protein** | **Beta_EpiScore_** | **P_EpiScore_** | **Beta_Measured_g_** | **P_Measured_g_** | **Olink Panel** |
| --- | --- | --- | --- | --- | --- |
| IL8 | -0.156 | 8.9E-06 | -0.056 | 1.2E-01 | Inflammation |
| VEGFA | -0.140 | 6.7E-05 | -0.087 | 1.6E-02 | Inflammation |
| MCP.3 | -0.142 | 5.6E-05 | -0.113 | 1.8E-03 | Inflammation |
| CDCP1 | -0.102 | 3.7E-03 | -0.187 | 2.1E-07 | Inflammation |
| CD244 | -0.086 | 1.4E-02 | -0.074 | 4.1E-02 | Inflammation |
| IL7 | -0.103 | 3.4E-03 | -0.037 | 3.0E-01 | Inflammation |
| OPG | -0.118 | 7.6E-04 | -0.126 | 4.4E-04 | Inflammation |
| LAP.TGF.beta.1 | -0.106 | 2.6E-03 | -0.086 | 1.8E-02 | Inflammation |
| uPA | -0.085 | 1.4E-02 | -0.072 | 4.3E-02 | Inflammation |
| IL6 | -0.165 | 2.5E-06 | -0.131 | 3.1E-04 | Inflammation |
| MCP.1 | -0.078 | 2.7E-02 | -0.038 | 2.9E-01 | Inflammation |
| CXCL11 | -0.102 | 3.7E-03 | -0.035 | 3.3E-01 | Inflammation |
| AXIN1 | -0.019 | 5.9E-01 | -0.035 | 3.3E-01 | Inflammation |
| TRAIL | -0.056 | 1.1E-01 | 0.036 | 3.2E-01 | Inflammation |
| CXCL9 | -0.083 | 1.9E-02 | -0.057 | 1.1E-01 | Inflammation |
| CST5 | -0.040 | 2.6E-01 | 0.044 | 2.3E-01 | Inflammation |
| OSM | -0.099 | 4.8E-03 | -0.041 | 2.6E-01 | Inflammation |
| CXCL1 | -0.111 | 1.6E-03 | -0.071 | 5.1E-02 | Inflammation |
| CCL4 | -0.112 | 1.4E-03 | -0.052 | 1.5E-01 | Inflammation |
| CD6 | -0.051 | 1.4E-01 | -0.046 | 2.0E-01 | Inflammation |
| SCF | -0.063 | 7.3E-02 | 0.057 | 1.2E-01 | Inflammation |
| IL18 | -0.062 | 7.6E-02 | -0.085 | 1.7E-02 | Inflammation |
| SLAMF1 | -0.112 | 1.2E-03 | -0.077 | 3.0E-02 | Inflammation |
| TGF.alpha | -0.095 | 6.8E-03 | -0.045 | 2.2E-01 | Inflammation |
| MCP.4 | -0.036 | 2.9E-01 | -0.029 | 4.2E-01 | Inflammation |
| CCL11 | -0.070 | 4.4E-02 | -0.060 | 9.6E-02 | Inflammation |
| TNFSF14 | -0.112 | 1.3E-03 | -0.067 | 6.1E-02 | Inflammation |
| FGF.23 | -0.079 | 2.5E-02 | -0.098 | 6.5E-03 | Inflammation |
| FGF.5 | -0.043 | 2.1E-01 | -0.074 | 3.8E-02 | Inflammation |
| MMP.1 | -0.019 | 5.9E-01 | -0.067 | 6.4E-02 | Inflammation |
| LIF.R | -0.055 | 1.2E-01 | -0.052 | 1.5E-01 | Inflammation |
| FGF.21 | -0.028 | 4.2E-01 | -0.148 | 3.9E-05 | Inflammation |
| CCL19 | -0.061 | 8.6E-02 | -0.076 | 3.5E-02 | Inflammation |
| IL.15RA | -0.087 | 1.4E-02 | -0.075 | 3.7E-02 | Inflammation |
| IL.10RB | -0.100 | 4.6E-03 | -0.091 | 1.2E-02 | Inflammation |
| IL.18R1 | -0.166 | 2.3E-06 | -0.134 | 2.0E-04 | Inflammation |
| PD.L1 | -0.113 | 1.2E-03 | -0.030 | 4.0E-01 | Inflammation |
| Beta.NGF | -0.109 | 2.0E-03 | -0.003 | 9.3E-01 | Inflammation |
| CXCL5 | 0.008 | 8.1E-01 | -0.017 | 6.3E-01 | Inflammation |
| TRANCE | 0.009 | 8.0E-01 | -0.013 | 7.2E-01 | Inflammation |
| HGF | -0.126 | 3.3E-04 | -0.112 | 2.0E-03 | Inflammation |
| IL.12B | -0.085 | 1.5E-02 | -0.014 | 6.9E-01 | Inflammation |
| MMP.10 | -0.050 | 1.5E-01 | -0.037 | 3.0E-01 | Inflammation |
| IL10 | -0.065 | 6.6E-02 | -0.043 | 2.4E-01 | Inflammation |
| CCL23 | -0.073 | 3.9E-02 | -0.016 | 6.6E-01 | Inflammation |
| CD5 | -0.099 | 4.8E-03 | -0.060 | 9.4E-02 | Inflammation |
| CCL3 | -0.142 | 5.2E-05 | -0.083 | 2.2E-02 | Inflammation |
| Flt3L | -0.125 | 3.5E-04 | -0.078 | 3.0E-02 | Inflammation |
| CXCL6 | -0.070 | 4.7E-02 | -0.033 | 3.6E-01 | Inflammation |
| CXCL10 | -0.091 | 9.8E-03 | -0.053 | 1.4E-01 | Inflammation |
| X4E.BP1 | -0.037 | 2.8E-01 | -0.073 | 4.3E-02 | Inflammation |
| SIRT2 | -0.031 | 3.8E-01 | -0.028 | 4.3E-01 | Inflammation |
| CCL28 | -0.031 | 3.8E-01 | -0.065 | 7.3E-02 | Inflammation |
| DNER | -0.018 | 6.2E-01 | -0.007 | 8.4E-01 | Inflammation |
| EN.RAGE | -0.091 | 1.0E-02 | -0.032 | 3.8E-01 | Inflammation |
| CD40 | -0.102 | 3.7E-03 | -0.067 | 6.4E-02 | Inflammation |
| FGF.19 | 0.003 | 9.3E-01 | -0.010 | 7.8E-01 | Inflammation |
| MCP.2 | -0.065 | 6.7E-02 | -0.075 | 3.8E-02 | Inflammation |
| CASP.8 | -0.074 | 3.5E-02 | -0.043 | 2.4E-01 | Inflammation |
| CCL25 | -0.046 | 1.9E-01 | -0.090 | 1.3E-02 | Inflammation |
| CX3CL1 | -0.049 | 1.7E-01 | -0.035 | 3.3E-01 | Inflammation |
| TNFRSF9 | -0.166 | 2.3E-06 | -0.060 | 9.9E-02 | Inflammation |
| NT.3 | -0.123 | 4.4E-04 | -0.013 | 7.1E-01 | Inflammation |
| TWEAK | -0.006 | 8.6E-01 | 0.013 | 7.1E-01 | Inflammation |
| CCL20 | -0.043 | 2.3E-01 | -0.100 | 5.5E-03 | Inflammation |
| ST1A1 | -0.005 | 8.9E-01 | 0.005 | 8.8E-01 | Inflammation |
| STAMBP | -0.038 | 2.8E-01 | -0.036 | 3.2E-01 | Inflammation |
| ADA | -0.081 | 2.1E-02 | -0.078 | 3.1E-02 | Inflammation |
| TNFB | -0.045 | 1.9E-01 | -0.059 | 9.7E-02 | Inflammation |
| CSF.1 | -0.124 | 4.0E-04 | -0.070 | 5.3E-02 | Inflammation |
| ADAM.22 | -0.072 | 6.5E-02 | Harris et al. Table S2 | | Neurology |
| ADAM.23 | -0.072 | 6.2E-02 | Harris et al. Table S2 | | Neurology |
| Alpha.2.MRAP | -0.072 | 6.3E-02 | Harris et al. Table S2 | | Neurology |
| BCAN | 0.031 | 4.3E-01 | Harris et al. Table S2 | | Neurology |
| Beta.NGF | -0.095 | 1.4E-02 | Harris et al. Table S2 | | Neurology |
| BMP.4 | -0.063 | 1.1E-01 | Harris et al. Table S2 | | Neurology |
| CADM3 | -0.040 | 3.1E-01 | Harris et al. Table S2 | | Neurology |
| CD200 | -0.043 | 2.7E-01 | Harris et al. Table S2 | | Neurology |
| CD200R1 | -0.099 | 1.1E-02 | Harris et al. Table S2 | | Neurology |
| CD38 | -0.086 | 2.6E-02 | Harris et al. Table S2 | | Neurology |
| CDH3 | -0.044 | 2.6E-01 | Harris et al. Table S2 | | Neurology |
| CDH6 | -0.079 | 4.2E-02 | Harris et al. Table S2 | | Neurology |
| CLEC10A | -0.050 | 2.0E-01 | Harris et al. Table S2 | | Neurology |
| CLEC1B | -0.038 | 3.3E-01 | Harris et al. Table S2 | | Neurology |
| CLM.1 | -0.058 | 1.4E-01 | Harris et al. Table S2 | | Neurology |
| CLM.6 | -0.122 | 1.7E-03 | Harris et al. Table S2 | | Neurology |
| CNTN5 | 0.027 | 4.9E-01 | Harris et al. Table S2 | | Neurology |
| CPA2 | -0.078 | 4.4E-02 | Harris et al. Table S2 | | Neurology |
| CPM | -0.154 | 7.0E-05 | Harris et al. Table S2 | | Neurology |
| CRTAM | -0.210 | 5.1E-08 | Harris et al. Table S2 | | Neurology |
| CTSC | -0.065 | 9.5E-02 | Harris et al. Table S2 | | Neurology |
| CTSS | -0.149 | 1.2E-04 | Harris et al. Table S2 | | Neurology |
| DDR1 | -0.101 | 9.1E-03 | Harris et al. Table S2 | | Neurology |
| Dkk.4 | -0.086 | 2.6E-02 | Harris et al. Table S2 | | Neurology |
| DRAXIN | -0.065 | 9.7E-02 | Harris et al. Table S2 | | Neurology |
| EDA2R | -0.150 | 1.0E-04 | Harris et al. Table S2 | | Neurology |
| EFNA4 | -0.150 | 1.0E-04 | Harris et al. Table S2 | | Neurology |
| EPHB6 | -0.080 | 3.9E-02 | Harris et al. Table S2 | | Neurology |
| EZR | -0.038 | 3.3E-01 | Harris et al. Table S2 | | Neurology |
| FcRL2 | -0.072 | 6.5E-02 | Harris et al. Table S2 | | Neurology |
| FLRT2 | -0.114 | 3.2E-03 | Harris et al. Table S2 | | Neurology |
| G.CSF | -0.106 | 6.2E-03 | Harris et al. Table S2 | | Neurology |
| gal.8 | -0.055 | 1.5E-01 | Harris et al. Table S2 | | Neurology |
| GCP5 | 0.026 | 5.1E-01 | Harris et al. Table S2 | | Neurology |
| GDF.8 | -0.031 | 4.3E-01 | Harris et al. Table S2 | | Neurology |
| GDNF | -0.068 | 8.0E-02 | Harris et al. Table S2 | | Neurology |
| GDNFR.alpha.3 | -0.065 | 9.6E-02 | Harris et al. Table S2 | | Neurology |
| GFR.alpha.1 | -0.097 | 1.3E-02 | Harris et al. Table S2 | | Neurology |
| GM.CSF.R.alpha | -0.016 | 6.8E-01 | Harris et al. Table S2 | | Neurology |
| GZMA | -0.182 | 2.3E-06 | Harris et al. Table S2 | | Neurology |
| IL.5R.alpha | -0.015 | 7.1E-01 | Harris et al. Table S2 | | Neurology |
| IL12 | -0.106 | 6.5E-03 | Harris et al. Table S2 | | Neurology |
| JAM.B | -0.113 | 3.6E-03 | Harris et al. Table S2 | | Neurology |
| KYNU | -0.086 | 2.7E-02 | Harris et al. Table S2 | | Neurology |
| LAIR.2 | -0.040 | 3.1E-01 | Harris et al. Table S2 | | Neurology |
| LAT | -0.020 | 6.1E-01 | Harris et al. Table S2 | | Neurology |
| LAYN | -0.096 | 1.4E-02 | Harris et al. Table S2 | | Neurology |
| LXN | -0.138 | 3.6E-04 | Harris et al. Table S2 | | Neurology |
| MANF | -0.047 | 2.2E-01 | Harris et al. Table S2 | | Neurology |
| MATN3 | -0.022 | 5.8E-01 | Harris et al. Table S2 | | Neurology |
| MDGA1 | -0.065 | 9.3E-02 | Harris et al. Table S2 | | Neurology |
| MSR1 | -0.179 | 3.3E-06 | Harris et al. Table S2 | | Neurology |
| N.CDase | -0.059 | 1.3E-01 | Harris et al. Table S2 | | Neurology |
| N2DL.2 | -0.128 | 9.2E-04 | Harris et al. Table S2 | | Neurology |
| NAAA | 0.028 | 4.7E-01 | Harris et al. Table S2 | | Neurology |
| NBL1 | -0.052 | 1.8E-01 | Harris et al. Table S2 | | Neurology |
| NCAN | -0.015 | 6.9E-01 | Harris et al. Table S2 | | Neurology |
| NEP | -0.096 | 1.3E-02 | Harris et al. Table S2 | | Neurology |
| NMNAT1 | 0.046 | 2.3E-01 | Harris et al. Table S2 | | Neurology |
| Nr.CAM | -0.116 | 2.8E-03 | Harris et al. Table S2 | | Neurology |
| NRP2 | -0.133 | 6.0E-04 | Harris et al. Table S2 | | Neurology |
| NTRK2 | -0.051 | 1.9E-01 | Harris et al. Table S2 | | Neurology |
| NTRK3 | 0.043 | 2.6E-01 | Harris et al. Table S2 | | Neurology |
| PDGF.R.alpha | -0.094 | 1.6E-02 | Harris et al. Table S2 | | Neurology |
| PLXNB1 | -0.089 | 2.2E-02 | Harris et al. Table S2 | | Neurology |
| PLXNB3 | 0.006 | 8.7E-01 | Harris et al. Table S2 | | Neurology |
| PRTG | -0.040 | 3.0E-01 | Harris et al. Table S2 | | Neurology |
| PVR | -0.134 | 5.3E-04 | Harris et al. Table S2 | | Neurology |
| RGMA | -0.047 | 2.2E-01 | Harris et al. Table S2 | | Neurology |
| RGMB | -0.086 | 2.7E-02 | Harris et al. Table S2 | | Neurology |
| ROBO2 | -0.091 | 1.9E-02 | Harris et al. Table S2 | | Neurology |
| RSPO1 | -0.084 | 3.1E-02 | Harris et al. Table S2 | | Neurology |
| SCARA5 | -0.105 | 6.8E-03 | Harris et al. Table S2 | | Neurology |
| SCARB2 | -0.143 | 2.3E-04 | Harris et al. Table S2 | | Neurology |
| SCARF2 | -0.036 | 3.5E-01 | Harris et al. Table S2 | | Neurology |
| sFRP.3 | -0.030 | 4.5E-01 | Harris et al. Table S2 | | Neurology |
| Siglec.9 | -0.162 | 2.8E-05 | Harris et al. Table S2 | | Neurology |
| SIGLEC1 | -0.182 | 2.5E-06 | Harris et al. Table S2 | | Neurology |
| SKR3 | -0.115 | 2.9E-03 | Harris et al. Table S2 | | Neurology |
| SMOC2 | -0.119 | 2.1E-03 | Harris et al. Table S2 | | Neurology |
| SMPD1 | -0.059 | 1.3E-01 | Harris et al. Table S2 | | Neurology |
| SPOCK1 | 0.040 | 3.0E-01 | Harris et al. Table S2 | | Neurology |
| THY.1 | -0.137 | 4.2E-04 | Harris et al. Table S2 | | Neurology |
| TMPRSS5 | -0.014 | 7.2E-01 | Harris et al. Table S2 | | Neurology |
| TN.R | 0.047 | 2.3E-01 | Harris et al. Table S2 | | Neurology |
| TNFRSF12A | -0.122 | 1.7E-03 | Harris et al. Table S2 | | Neurology |
| TNFRSF21 | -0.063 | 1.1E-01 | Harris et al. Table S2 | | Neurology |
| UNC5C | -0.119 | 2.1E-03 | Harris et al. Table S2 | | Neurology |
| VWC2 | -0.087 | 2.5E-02 | Harris et al. Table S2 | | Neurology |
| WFIKKN1 | 0.020 | 6.0E-01 | Harris et al. Table S2 | | Neurology |

**Table S13.** Age at MRI scan- and sex-adjusted linear regression associations between the Epigenetic *g* Score (EpiScore) and measured *g* score and traits associated with global neuroimaging outcomes in the Lothian Birth Cohorts 1936. All continuous variables are standardised to mean 0 and variance 1.

| **Trait** | **β_EpiScore_** | **P_EpiScore_** | **β_Measured g_** | **P_Measured g_** |
| --- | --- | --- | --- | --- |
| Total brain volume | 0.15 | 3.8E-06 | 0.25 | 4.4E-14 |
| Grey matter volume | 0.14 | 4.3E-05 | 0.25 | 6.9E-14 |
| Normal appearing white matter volume | 0.15 | 1.3E-05 | 0.28 | 1.9E-15 |
| Global fractional anisotropy | 0.003 | 8.6E-01 | 0.11 | 4.6E-02 |
| Global mean diffusivity | 0.08 | 1.3E-01 | -0.08 | 1.2E-01 |
