# Supplementary material for: Blood-based epigenome-wide analyses of cognitive abilities
Source: Genome Biol. 2022 Jan 17;23:26. doi: 10.1186/s13059-021-02596-5 (PMC8762878; doi:10.1186/s13059-021-02596-5)
Supplement: Supplementary file 2 — Additional file 2: Supplementary Figures 1-5. Figure S1: Plot of the epigenetic smoking variable (EpiSmoker) against self-reported smoking status (current, former, never) in Generation Scotland (n=9,162). Figure S2: Median effects observed for DNA methylation probes with posterior inclusion probabilities (PIPs) > 0.8 across four cognitive tests and two composite measures (no probes identified for logical memory). Figure S3: Scatter Plot of Epigenetic g Score by Measured g Score in the Lothian Birth Cohort 1936 (LBC1936) and the Lothian Birth Cohort 1921 (LBC1921). Figure S4: Regional cortical volume regressed against measured g (left) and EpiScore g (middle), colours denote the magnitude (T-maps; top, A-B) and significance (Q values; bottom, D-E) of the negative associations between cognitive measures and brain cortical volume. Panel (C) shows the percentage attenuation for the significant associations between EpiScore and cortical volume when also controlling for measured g. (F) shows the spatial extent overlap (green) in cortical loci that exhibit FDR-corrected unique associations. Figure S5: Regional cortical thickness regressed against measured g (left) and EpiScore g (middle), colours denote the magnitude (T-maps; top, A-B) and significance (Q values; bottom, D-E) of the negative associations between cognitive measures and brain cortical thickness. Panel (C) shows the percentage attenuation for the significant associations between EpiScore and cortical thickness when also controlling for measured g. (F) shows the spatial extent overlap (green) in cortical loci that exhibit FDR-corrected unique associations. [file 13059_2021_2596_MOESM2_ESM.docx]

**Figure S1:** Plot of the epigenetic smoking variable (EpiSmoker) against self-reported smoking status (current, former, never) in Generation Scotland (n=9,162).


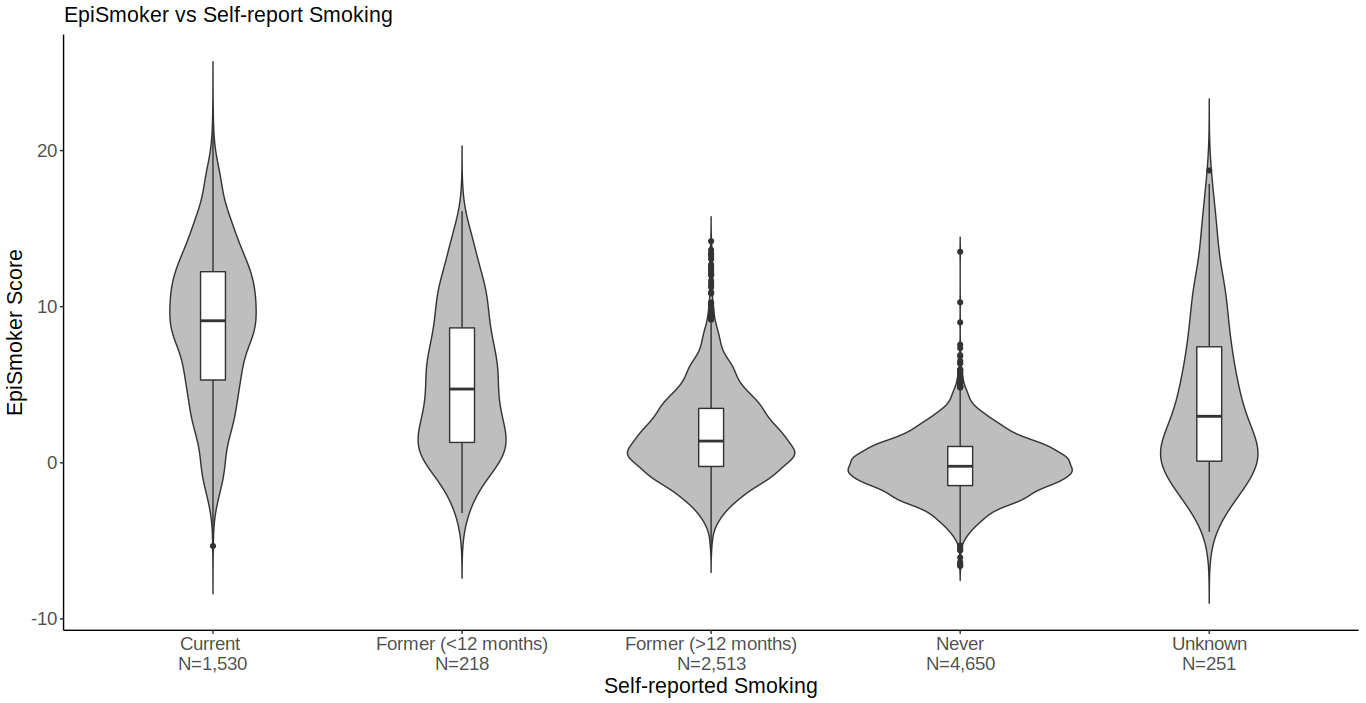


**Figure S2:** Median effects observed for DNA methylation probes with posterior inclusion probabilities (PIPs) > 0.8 across four cognitive tests and two composite measures (no probes identified for logical memory). Thick horizontal lines represent the 5^th^ and 95^th^ percentiles; thin lines represent 2.5^th^ and 97.5^th^ percentiles. Left panel displays summaries for all traits, right panel displays summaries for traits where PIP > 0.8.


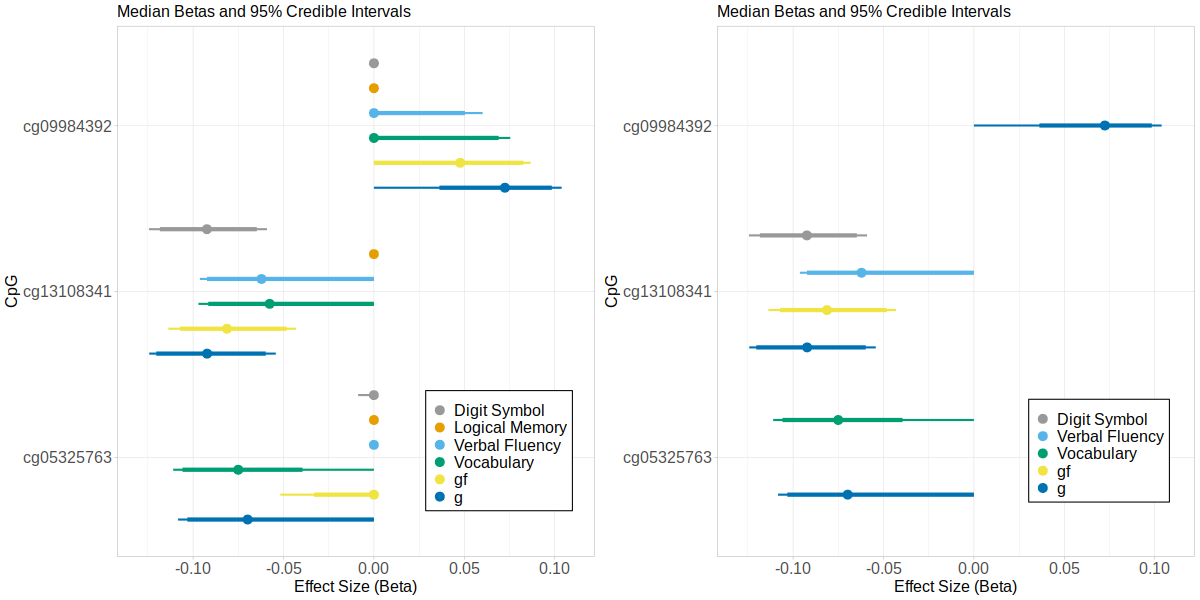


**Figure S3.** Scatter Plot of Epigenetic *g* Score by Measured *g* Score in the Lothian Birth Cohort 1936 (LBC1936) and the Lothian Birth Cohort 1921 (LBC1921).


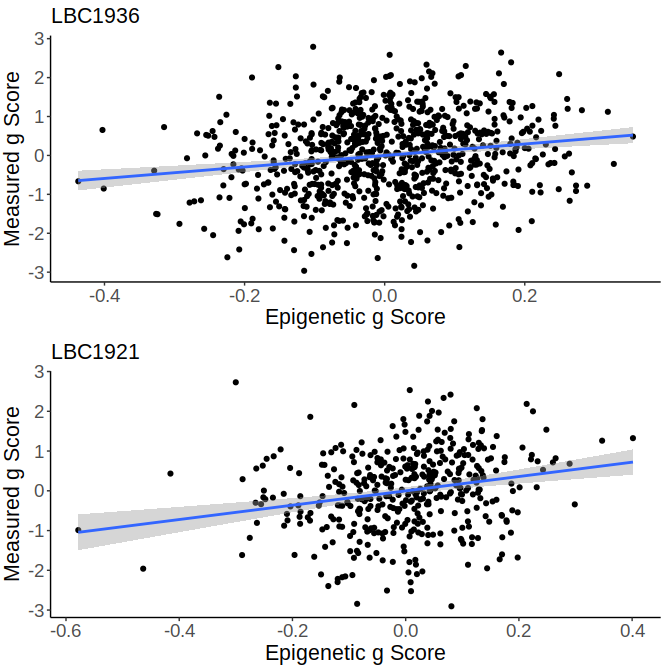


**Figure S4.** Regional cortical volume regressed against measured *g* (**left**) and EpiScore *g* (**middle**), colours denote the magnitude (T-maps; top, **A-B**) and significance (Q values; bottom, **D-E**) of the negative associations between cognitive measures and brain cortical volume. Panel (**C**) shows the percentage attenuation for the significant associations between EpiScore and cortical volume when also controlling for measured *g*. (**F**) shows the spatial extent overlap (green) in cortical loci that exhibit FDR-corrected unique associations.

**Figure S5.** Regional cortical thickness regressed against measured g (**left**) and EpiScore *g* (**middle**), colours denote the magnitude (T-maps; top, **A-B**) and significance (Q values; bottom, **D-E**) of the negative associations between cognitive measures and brain cortical thickness. Panel (**C)** shows the percentage attenuation for the significant associations between EpiScore and cortical thickness when also controlling for measured *g*. (**F**) shows the spatial extent overlap (green) in cortical loci that exhibit FDR-corrected unique associations.

**
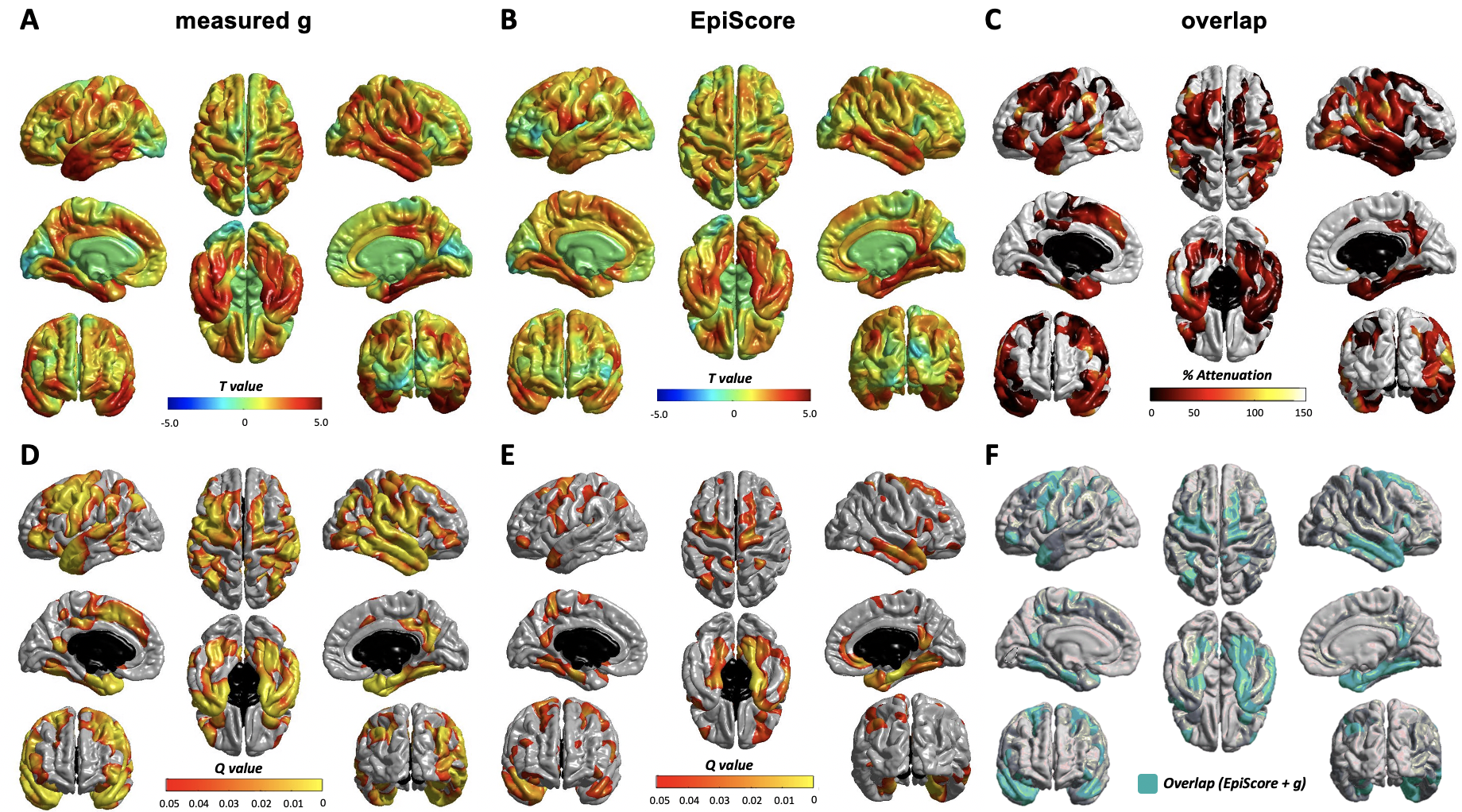
**
