# Supplementary material for: Blood-based epigenome-wide analyses of cognitive abilities
Source: Genome Biol. 2022 Jan 17;23:26. doi: 10.1186/s13059-021-02596-5 (PMC8762878; doi:10.1186/s13059-021-02596-5)
Supplement: Supplementary file 3 — Additional file 3. Online Imaging Methods. [file 13059_2021_2596_MOESM3_ESM.docx]

**Online Methods**

*Online Imaging Methods*

Structural and diffusion tensor (DTI) MRI acquisition and processing in LBC1936 were performed at Wave 2 (age 73 years) according to an open-access protocol [42]. A 1.5 T GE Signa HDx clinical scanner (General Electric, Milwaukee, WI, USA) was used to collect structural T1- (voxel size = 1 × 1 × 1.3 mm), T2- (voxel size = 1 × 1 × 2 mm, T2*- (voxel size = 1 × 1 × 2 mm), and FLAIR-weighted images (voxel size = 1 × 1 × 4 mm). Diffusion MRI protocol consisted of a single-shot spin-echo echo-planar diffusion-weighted sequence. Diffusion-weighted volumes (b = 1000 s mm−2) were acquired in 64 non-collinear directions, with seven T2-weighted volumes (b = 0 s mm−2), with 72 contiguous axial slices, and an acquisition matrix of 128 × 128 and 2-mm isotropic voxels. Total brain, grey matter, and normal-appearing white matter (NAWM) volumes were calculated using a semi-automated multi-spectral fusion method [43]. Intracranial volume was determined semi-automatically using Analyze 11.0^TM^. White matter microstructural parameters fractional anisotropy (FA) and mean diffusivity (MD) were derived for 12 major tracts of interest: corpus callosum genu and splenium, bilateral frontal cingulum, arcuate, uncinate and superior longitudinal fasciculi and bilateral anterior thalamic radiation. The FA and MD values from each of the 12 tracts were the weighted average of the diffusion values contained within the resultant tract map. General measures of FA and MD (gFA and gMD) were the extracted scores from the first unrotated principal component, accounting for 37% and 41% of the variance in tract FA and MD, respectively. Cortical reconstruction and segmentation was performed using FreeSurfer v5.1 on T1-weighted volumes. QC involved visually assessing each image output for segmentation and parcellation errors, which were then corrected manually; segmentations with errors that could not be corrected were excluded. Participants were then excluded if they had self-reported history of dementia or signs of cognitive impairment (Mini mental state examination score < 24/30); after exclusions, a total of 590 participants had complete cognitive, epigenetic and global neuroimaging data, and of these, 551 participants had complete and vertex-wise neuroimaging data. Localized associations between cognitive measures and vertex-wise cortical volume and thickness were performed using linear regression, controlling for age, sex, and Intracranial volume (ICV). The SurfStat MATLAB toolbox (http://www.math.mcgill.ca/keith/surf stat) for Matrix Laboratory R2012a (The MathWorks, Inc., Natick, MA, USA) was used to carry out analyses. Statistical maps of association magnitude and valence (*t*-maps) and significance (q-maps; p-values corrected for multiple comparisons using a false discovery rate (FDR) with a q-value of 0.05 across all 327,684 vertices on the cortical surface) were presented.
