# Supplementary material for: Blood-based epigenome-wide analyses of cognitive abilities
Source: Genome Biol. 2022 Jan 17;23:26. doi: 10.1186/s13059-021-02596-5 (PMC8762878; doi:10.1186/s13059-021-02596-5)
Supplement: Supplementary file 4 — Additional file 4. Review history. [file 13059_2021_2596_MOESM4_ESM.docx]

Review History

**First round of review**

**Reviewer 1**

**Are you able to assess all statistics in the manuscript, including the appropriateness of statistical tests used?**

**Yes**

**Comments to author:**

The authors conducted a Bayesian variance component analysis and EWAS in a large cohort on cognitive function assessed via principal component analysis. Subsequently, an epigenetic score (EpiScore) was estimated from the EWAS and tested in two independent cohorts. Additional association analyses of the EpiScore in comparison with measured cognitive function on protein levels, global brain imaging metrics, and regional cortical volumes was conducted.
The analyses are generally sound, and the results interesting. However, several issues need to be addressed, which are mainly related to the description of the association models, adjustment for potential confounders, and interpretation of the results.

Specific comments:

More details on the EWAS association analysis need to be provided, i.e. was the correlation of phenotypic and DMAm data performed by using residuals? How was the white blood cell type composition taken into account in the EWAS and other DNAm-related analyses including the EpiScore?

According to the methods, the DNAm data were corrected for batch, age, sex and epigenetic smoking. Does it mean that the EpiScore was adjusted for smoking, but not for BMI? As a consequence, could the results of Table S8 be confounded by BMI (i.e. in LBC1936)? I suggest to take BMI as a potential confounder of the relationship between DMAm and cognitive phenotypes into account in the analyses and interpretation of the results.

A table providing the cohort summary of the two LBC cohorts with sex, age and the traits included in Tables S8 and S10 (for LBC1936) should be included.

How many CpGs were included in the EpiScore, and on which criterion were they selected (were only the three lead sites with PIP>0.8 used)? How were the "mean CpG weights" defined that were used for multiplication in the EpiScore calculation (Additional File 3, Statistical analysis)?

Do the authors have an explanation why the EpiScore outperformed the measured g in Table S8? Furthermore, there seems to be a significance criterion missing for the results in Table S8, although p-values are provided.

How was the very low p-value cutoff of p<3.6E-8 for the EWAS catalog lookup selected? E.g., cg24503796 seems to have a highly significant association with smoking in the catalog (p=9.9E-8), but did not pass the inclusion criteria for Table S6. In any case, this association deserves a discussion, taking into account that the DNAm in the current study was corrected for (epigenetic) smoking.

The Discussion is generally very short, and from my point of view it lacks interpreting several of the findings. E.g., what are the conclusions of the OLINK analyses? How could (at least) the two overlapping proteins IL-6 and FGF21 of the OLINK analyses (EpiScore and measured g) be functionally related to cognitive abilities? What are the limitations of the current study (using blood cells for the EWAS, using the g score as a surrogate for cognitive function, etc.)?

According to the Additional File 3 (Statistical analysis), the polygenic score for cognitive ability was derived from all possible SNPs (GWAS P<1) in a UK Biobank GWAS. Is the "P<1" a typo, or why were the GWAS results with P=1 excluded? On the other hand, P<1 seems too inclusive for a PRS.

The section "Statistical Analyses" in the Additional File 3 should be divided into meaningful subsections. Describing the different analyses in the currently very long single section is hard to read.

Please re-phrase the first sentence of the "Statistical Analysis" as it is not clear what "DNA methylation-based heritability" means. I assume, it should read something like "...to estimate the heritability and DNA methylation-based explained variance of the cognitive traits."

The colors of the dots in Figure 1 panels C and D are nice, but they should somehow be assigned to the protein names (e.g. via Table S9) to make the results more meaningful.

The panel letters in the Figure 1 panels are missing.

**Reviewer 2**

**Are you able to assess all statistics in the manuscript, including the appropriateness of statistical tests used?**

Yes

**Comments to author:**

The authors present an interesting assessment of the architecture of cognitive function, using a methodology adapted from GWAS hey have previously applied to other complex traits. If you are familiar with the Bayesian method to quantify the amount of variation explained by genetic and epigenetic effects, then you can follow the results but I believe many readers are not. Therefore, some additional explanation on the nuances of the analysis and how to interpret the results would be highly beneficial. Currently it reads like a list of statistics, with little linking different analyses or explaining the purpose of them. For example, the last sentence of the second results paragraph in needs more introduction and the second clause in this sentence reads more like a figure legend than a narrative. I also some think some discussion on the interplay of genetics and epigenetics and why you would assess both simultaneously.

I have only minor concerns regarding the way the analysis was performed but given the novelty of this method in the context of epigenetics, I have a number of queries that I was hoping the authors could explain. They do not need to incorporate all of this into a revised version, but I imagine some of would be of interest to the broader epigenetic epidemiology field to enable them to properly digest what is being presented.

Specific comments below

1) Is it surprising that DNAm can explain such a high proportion of the variance in a trait given that the EPIC array only captures ~3% of CpGs? We know SNP chips capture ~ 95% of common variation, so large proportions of variance make some sense (although I am ignoring rare variants) but I do not believe that the EPIC array is comparably comprehensive and therefore struggle to believe the numbers.

2) Does it make sense that blood based DNA methylation profiles would capture such a large proportion of variation of arguably a brain trait? Is it feasible that a blood based biomarker would ever be a sufficiently precise predictor (particularly given the statistics presented for a training sample of 100,000 where R2 = 29%).

3) Of the ~840,000 sites on the EPIC array, the majority of these do not vary (at least within a tissue/cell type) and are effectively 0 or 100% methylated. These are unlikely to be truly associated to the outcome, are estimated more imprecisely and more likely to result in false positive associations (due to smaller variance). How does the result change if these are excluded? What is the value of retaining these in the analysis?

4) When the analysis concludes that "The majority of the variance was accounted for by CpG sites with small effects" is this interpretation limited to the sites directly measured by the array, or does it tell us about sites not assayed.

5) Given the potential of reverse causality in epigenetic epidemiology, how should one interpret these results? Surely, these are not all causal sites. If they are not, are they consequences or result from confounders? Do these options not violate the assumptions of the statistics? Here I am questioning whether GWAS methods can simply be lifted and applied to EWAS data without the interpretation being given adequate thought.

6) The lack of shared signal between the genetic and epigenetic components is particularly interesting. Could these be interpreted as the EpiScore reflecting more environmental risk factors of cognition? Does this EpiScore correlate with EpiScore for smoking, alcohol or obesity with the author's state in the introduction are risk factors for cognitive decline.

7) Given the broad age range in the dataset, there is a huge variation in where the individuals are on their lifelong cognitive trajectory. For some participants their g score will reflect their peak cognitive state, other older individual may be undergoing cognitive decline albeit not to clinical levels. What I am alluding to here is heterogeneity in the definition of the phenotype and risk factors that underlie an individual's cognitive state at that stage of their life. How might this influence the analysis?

8) In this statement:
Based on a training sample size of 10,000 and assuming 100,000 CpGs affect the trait, with a DNAm variance components estimate of 41.6%, we would expect a DNAm prediction R2 value of about 4.0% (following Formula 1 from [13]).
Where does the number of 100,000 CpGs come from? Are the authors suggesting that because these statistics fit the pattern observed data that is the total number of CpGs associated with cognitive function? The fact that in what is a very large sample by EWAS standards they only report three is somewhat disappointing.

9) How do we interpret the epiScore for cognition given it potentially captures some of the risk factors influencing cognitive function? Indeed the authors note these themselves in the Introduction. "However, blood-based 52 DNA methylation predictors of cognitive function itself, rather than its known correlates, may 53 index a wider range of risk factors for neurodegeneration." Can we then really call it a biomarker of cognition?

10) What does it mean that all the three lead cognition CpGs are associated with age? I find this a little concerning given the age range in your cohort. I know you regressed age out, and looked the addition of an age squared term, but if the relationship between cognition and age is non-linear, then the residuals will still be associated with age. Bias may also be introduced if there are outliers in either the g or the age distributions.

11) The SNP based heritability is reported as 37.9% with a 95% credible interval of [18,3% - 25.9%] how can the estimate not be included within the credible interval? Assuming the credible interval is correct, and the estimate is a typo, the precision for the SNP heritability is tighter than for the epigenetic estimate. Why is this? I assume they were performed on the same sample size.

12) I disagree that the estimates from the linear mixed models in whole cohort vs the single batch are similar, one is almost 50% bigger than the other. Surely, you would expect them to be within the SE (or ~2xSE). Does this suggest some bias/differences in the datasets? What's more, what units are the SE? Are they %, if so they are tiny suggesting the estimates are extremely accurate and further highlighting how different the estimates are.

13) I couldn't find anywhere in the manuscript for the 2 LBC cohorts the age at which cognition was assessed.

14) In table S2 what is the difference between g and gf?

**Reviewer 3**

**Are you able to assess all statistics in the manuscript, including the appropriateness of statistical tests used?**

Yes

**Comments to authors:**

The authors conducted analysis to understand associations between cognitive function and blood-based methylation measures using a large cohort of European ancestries. The biggest issue here is, this paper feels like a big data dump and need work in carefully laying out the rationale behind the conducted work.

1. The description of the "Statistical analysis" in the supplement file 3 is not organized not is it very clear. There is no rationale under any of the analysis done. The paper feels like a big data dump. The authors may want to have several sub-sections to summarize different types of analysis first with each sub-section having clear rationale behind. The authors can then provide details of the analyses within each sub-section. It is currently all blended together with no rationale in one gigantic paragraph of about 5 pages long.

2. There should also be details on the cohort in Online Methods instead of referring readers to several publications. What is the timeline of cognitive function measures and DNA methylation samples? Concurrent?

3. The samples are from two sets "5,200 related individuals from Set 1, and 4,588 genetically unrelated individuals from Set 2". It is important to clarify 1) what kind of relatedness are in Set 1, 2) if analyses using Set 1 have taken care of the relatedness. These should be clarified in "Statistical analysis".

4. Please provide details on how "predicted epigenetic scores" were obtained.

5. For QC steps, please provide number of CpGs removed at each step and number of subjects removed at each step clearly.

6. Much of the description of the statistical analysis is not clear, for example, the following description of analysis is not clear: "Linear regression was used to test for an association between the predicted epigenetic score (predictor) and measured general cognitive ability (outcome), in models adjusting for age, sex, and the polygenic score. Age- and sex-adjusted linear regression models were used to relate the Epigenetic Score (EpiScore) for g and the measured g score (predictors) with common risk factors (outcomes) for cognitive decline, frailty, and dementia: body mass index (BMI in kg/m2); year of education; self-reported smoking (ever versus never); self-reported weekly units of alcohol; self-reported high blood pressure (yes/no); lung function (forced expiratory volume in one second) adjusted for age, sex, and height; time taken to walk six metres (seconds); socioeconomic deprivation (Scottish Index of Multiple Deprivation in LBC1936 and social grades based on highest reached occupation [21] in LBC1921); and depression (HADS-D total from the Hospital Anxiety and Depression questionnaire) [22, 23, 24]. BMI, six metre walk time, and units of alcohol were log transformed to reduce skew - a constant of one was added to the alcohol units prior to transforming." Please rewrite these sentences. It is not clear what has been done.

7. When using BayesR+ package, it is important to also describe statistical methods used instead of just referring to "the package has been used".

8. Please describe in details the statistical method with steps and data used to generate each table, for example, for Table S2, it is currently described as "Variance components analyses indicated that 41.6% [95% Credible Interval 31.0%, 53.0%] of variance in g was explained by all DNA methylation probes". Please describe clearly 1) statistical approach and steps, 2) data that generated these proportions and the confidence interval.

9. There are many places that were described loosely, for example, in the following sentence "sensitivity analysis using a linear mixed model approach [5] with genetic and epigenetic relationship matrices yielded near identical results (43.4% (SE 0.03));", it is not clear what "genetic and epigenetic relationship matrices" are, and how the matrices are used to generate "identical results", and what is the aim of this sensitivity analysis.

**Authors Response**

**Point-by-point responses to the reviewers’ comments:**

Reviewer #1: The authors conducted a Bayesian variance component analysis and EWAS in a large cohort on cognitive function assessed via principal component analysis. Subsequently, an epigenetic score (EpiScore) was estimated from the EWAS and tested in two independent cohorts. Additional association analyses of the EpiScore in comparison with measured cognitive function on protein levels, global brain imaging metrics, and regional cortical volumes was conducted.
The analyses are generally sound, and the results interesting. However, several issues need to be addressed, which are mainly related to the description of the association models, adjustment for potential confounders, and interpretation of the results.

***Response:*** *We thank the reviewer for their positive comments and have provided a point-by-point response to the comments below.*

Specific comments:

More details on the EWAS association analysis need to be provided, i.e. was the correlation of phenotypic and DMAm data performed by using residuals? How was the white blood cell type composition taken into account in the EWAS and other DNAm-related analyses including the EpiScore?

***Response:*** *We appreciate the reviewer’s request for clarification on these points and have updated the manuscript accordingly. Further details have been added to describe how the DNA methylation data and cognitive phenotypes were processed prior to analysis.*

*Results: Paragraph 1:*

*“Prior to running the main analyses, the cognitive phenotypes were pre-corrected for four covariates: age, sex, BMI and an epigenetic smoking score [17]. The DNA methylation data were corrected for batch, age, sex and epigenetic smoking, which all have pervasive effects on DNAm levels. Residuals from these linear regression models were taken forward for the primary analyses.”*

*In relation to white blood cell proportions in the EWAS, we have shown that BayesR+ accounts for structural effects in the data, such as sample relatedness and cell proportions (in the same Generation Scotland cohort), thereby negating the requirement for such pre-corrections. We now note this in the manuscript.*

*Results: Paragraph 2:*

*“To determine the proportion of variance in g that can be explained by all CpG sites on the DNAm array and to identify individual CpGs associated with g, we conducted a Bayesian penalised regression and Gaussian mixture-based variance partitioning analysis using BayesR+ software. BayesR+ has been shown to implicitly control for white cell proportions, which are typically estimated from the DNAm data, related participants, and other unknown confounders [6]. Three mixture distributions were specified, corresponding to possible small, medium, and large effect sizes for the CpGs (explaining 0.01%, 0.1%, and 1% of the variance, respectively).”*

*We also now present the correlation between the cognitive EpiScore and measured white cell counts (neutrophils, lymphocytes, monocytes, eosinophils, and basophils) in the LBC1921 and LBC1936 studies. The largest absolute Pearson correlation is 0.072 (all P-values ≥0.050), indicating minimal evidence of confounding from white cell proportions.*

*Results: Paragraph 5:*

*“The resulting cognitive EpiScores showed very weak correlations (absolute Pearson r ≤ 0.072; all P-values ≥0.050) with measured white blood cell counts in both LBC1921 and LBC1936 (****Additional File 1, Table S9****).”*

According to the methods, the DNAm data were corrected for batch, age, sex and epigenetic smoking. Does it mean that the EpiScore was adjusted for smoking, but not for BMI? As a consequence, could the results of Table S8 be confounded by BMI (i.e. in LBC1936)? I suggest to take BMI as a potential confounder of the relationship between DMAm and cognitive phenotypes into account in the analyses and interpretation of the results.

***Response:*** *We thank the reviewer for raising this point. As noted above, we have clarified the pre-correction steps for both the cognitive and DNAm phenotypes. Whereas the cognitive phenotypes were pre-corrected for age, sex, BMI and smoking, the DNAm data were pre-corrected for age, sex, batch and smoking. The methylation signals are therefore only capturing non-BMI-related variance in the cognitive outcomes. However, as is clear from Figure 1A, the EpiScore does associate with both BMI and smoking patterns in LBC1936. This is possibly due to residual confounding or shared variance between BMI, other cognitive correlates, and the EpiScore in LBC1936 where the cognitive phenotype was only adjusted for age and sex. We also now included a supplementary table that presents a multiple regression of all covariates on measured cognitive function in LBC1936. The incremental R2 upon the addition of the EpiScore is 0.8%. Interestingly, neither BMI nor smoking are significant predictors in the model.*

*Results: Paragraph 7:*

*“A multiple regression model in LBC1936 with all covariates as predictors of measured g is presented in* ***Additional File 1, Table S11****. Upon addition of the EpiScore to the model, the incremental R^2^ estimate was 0.8% (P=1.8x10^-3^).”*

*Discussion: Paragraph 4*

*“The EpiScore can therefore reflect consequences of processes linked to cognitive health. This is evident by the attenuation of the incremental R^2^ estimate from 3.4% to 0.8% in a multiple regression model that included age and sex plus ten covariates that are established correlates/risk factors for cognitive ability and cognitive decline. The correlation with these risk factors and the independent contribution of the EpiScore from the polygenic score for prediction of measured g emphasises the environmental variance being captured by the EpiScore.”*

A table providing the cohort summary of the two LBC cohorts with sex, age and the traits included in Tables S8 and S10 (for LBC1936) should be included*.*

***Response:*** *We thank the reviewer for this suggestion and have added a table of demographics for the Lothian Birth Cohorts to* ***Additional File 1, Table S8****.*

How many CpGs were included in the EpiScore, and on which criterion were they selected (were only the three lead sites with PIP>0.8 used)? How were the "mean CpG weights" defined that were used for multiplication in the EpiScore calculation (Additional File 3, Statistical analysis)?

***Response:*** *The EpiScore was constructed under a “polyepigenetic” model, and therefore included all CpGs which fed into the BayesR analysis (i.e. those passing quality control in both Generation Scotland datasets). The mean posterior weights were calculated from the effects of each CpG on general cognitive ability (g) over 1,000 iterations in BayesR. We have clarified this in the text.*

*Results: Paragraph 5:*

*“A weighted linear Epigenetic Score (EpiScore) for g that included all CpG sites in the EWAS was applied to two independent cohorts to determine the proportion of variance in g that can be explained by a single predictor variable. The weights for each CpG were the mean posterior effect sizes from the EWAS model of g. These weights were applied to CpGs in two independent studies, The Lothian Birth Cohort 1936 (LBC1936) and The Lothian Birth Cohort 1921 (LBC1921)”*

Do the authors have an explanation why the EpiScore outperformed the measured g in Table S8? Furthermore, there seems to be a significance criterion missing for the results in Table S8, although p-values are provided.

***Response:*** *The results of Table S8 for LBC1936 are also presented in Figure 1B (copied below, along with a version for LBC1921). The EpiScore only outperformed the measured g score in its association with alcohol although there is very little difference in the point estimates. We have added SEs to Table S8 (now Table S10) and include below the results plot for LBC1921 to illustrate that, where the EpiScore point estimate is larger than the measured g association, the differences in effects are minimal. We did not interpret the statistical significance of these findings but have highlighted the nominally significant (P<0.05) results in the updated files.*

How was the very low p-value cutoff of p<3.6E-8 for the EWAS catalog lookup selected? E.g., cg24503796 seems to have a highly significant association with smoking in the catalog (p=9.9E-8), but did not pass the inclusion criteria for Table S6. In any case, this association deserves a discussion, taking into account that the DNAm in the current study was corrected for (epigenetic) smoking.

***Response*** *Regarding the probe listed in the table (cg24503796), we thank the reviewer for drawing this to our attention. This was inadvertently carried over from a previous version of the EWAS catalog lookup, in which a less stringent probe set was queried. This should instead be cg05325763. We have updated the lookup table (Table S6) accordingly and apologise for this error. There were no associations in the EWAS catalog for cg05325763 at P<3.6x10^-8^*

*The use of 3.6 x 10^-8^ as a threshold for genome-wide significance is based on Saffari et al. (2018), who proposed this as a genome-wide estimate for studies of DNA methylation on the Illumina 450k array. We have clarified this in the manuscript:*

*Results: Paragraph 4:*

*“For the three lead CpG sites we queried the EWAS catalog (accessed 5 April 2021) for associations with other traits at a previously defined epigenome-wide significance threshold of P<3.6x10^-8^ (****Additional File 1, Table S6****) [11].”*

The Discussion is generally very short, and from my point of view it lacks interpreting several of the findings.  E.g., what are the conclusions of the OLINK analyses? How could (at least) the two overlapping proteins IL-6 and FGF21 of the OLINK analyses (EpiScore and measured g) be functionally related to cognitive abilities? What are the limitations of the current study (using blood cells for the EWAS, using the g score as a surrogate for cognitive function, etc.)?

***Response*** *We acknowledge the brevity of the discussion and have expanded it – we have asked the Editor to change the format of the manuscript from a Short Report to a Research article to facilitate this.*

*There is an extensive discussion of the neurology-related proteins associations with cognitive function in the Harris et al. paper that we cite. We have added new text to comment on the two inflammation-related proteins. Please note the overlap corresponds to IL.18R1, rather than FGF21, which was listed in the main text in error (see* ***Additional File 1, Table S12*** *[formerly* ***Table S9****] for the original summary statistics). The manuscript has been amended to state this. IL-6 is a well-established measure of inflammation and has been linked to cognitive function across the cognitive epidemiology literature. IL18R1 is subunit of the receptor complex for IL18, which mediates neuroinflammation and neurodegeneration in the central nervous system.*

*Discussion: Paragraph 2:*

*“Whereas many of the associations with measured cognitive ability in LBC1936 have been studied and discussed in detail previously (e.g., neurology proteins, brain MRI, health and lifestyle variables [16, 17]), we present for the first time the associations with inflammatory proteins. The EpiScore and measured g both associated with levels of IL-6 (a well-established correlate of cognitive ability [18]) and IL18.R1, a subunit of the IL18 receptor which with IL18 is associated with neuroinflammatory and neurodegenerative processes [19].*

According to the Additional File 3 (Statistical analysis), the polygenic score for cognitive ability was derived from all possible SNPs (GWAS P<1) in a UK Biobank GWAS. Is the "P<1" a typo, or why were the GWAS results with P=1 excluded? On the other hand, P<1 seems too inclusive for a PRS.

***Response:*** *Thank you for the query. The original sentence is correct. We took all possible SNPs for the construction of the polygenic score. Previous studies using the same UK Biobank data for polygenic prediction of cognitive ability have found minimal differences across P-value thresholds for constructing scores.*

[*https://www.nature.com/articles/s41380-017-0001-5*](https://www.nature.com/articles/s41380-017-0001-5)

*Although P-values of exactly 1 are not possible unless the outcome is predicted perfectly by the null model – which is not the case in GWAS – we have altered the manuscript to read P≤1 to avoid possible confusion.*

The section "Statistical Analyses" in the Additional File 3 should be divided into meaningful subsections. Describing the different analyses in the currently very long single section is hard to read.

***Response:*** *We agree with the reviewer and have re-worked the statistical analysis section into shorter, labelled sections.*

Please re-phrase the first sentence of the "Statistical Analysis" as it is not clear what "DNA methylation-based heritability" means. I assume, it should read something like "...to estimate the heritability and DNA methylation-based explained variance of the cognitive traits."

***Response:*** *The sentence has been re-phrased as follows:*

*Methods: Variance components analysis and EWAS using BayesR+:*

*“BayesR+ was used for the EWAS and to estimate the proportion of variance in cognitive traits explained by genetics and DNA methylation.”*

The colors of the dots in Figure 1 panels C and D are nice, but they should somehow be assigned to the protein names (e.g. via Table S9) to make the results more meaningful.

***Response:*** *We appreciate this feedback from the reviewer. However, we believe colour-coding might add unnecessary complexity to the plots. We instead propose labelling the green-coloured points (i.e. significantly associated with both EpiScore and measured g) in the plot area and have provided versions of these figures for consideration:*

The panel letters in the Figure 1 panels are missing.

***Response:*** *We thank the reviewer for highlighting this omission. The resubmitted version of the manuscript now contains panel letters in Figure 1.*

Reviewer #2:
The authors present an interesting assessment of the architecture of cognitive function, using a methodology adapted from GWAS hey have previously applied to other complex traits. If you are familiar with the Bayesian method to quantify the amount of variation explained by genetic and epigenetic effects, then you can follow the results but I believe many readers are not.  Therefore, some additional explanation on the nuances of the analysis and how to interpret the results would be highly beneficial. Currently it reads like a list of statistics, with little linking different analyses or explaining the purpose of them. For example, the last sentence of the second results paragraph in needs more introduction and the second clause in this sentence reads more like a figure legend than a narrative. I also some think some discussion on the interplay of genetics and epigenetics and why you would assess both simultaneously.

I have only minor concerns regarding the way the analysis was performed but given the novelty of this method in the context of epigenetics, I have a number of queries that I was hoping the authors could explain. They do not need to incorporate all of this into a revised version, but I imagine some of would be of interest to the broader epigenetic epidemiology field to enable them to properly digest what is being presented.

***Response:*** *We thank the reviewer for their helpful comments. We have made major edits to the paper to improve the flow of the results section with additional information on the methods and rationale.*

Specific comments below

1) Is it surprising that DNAm can explain such a high proportion of the variance in a trait given that the EPIC array only captures ~3% of CpGs?  We know SNP chips capture ~ 95% of common variation, so large proportions of variance make some sense (although I am ignoring rare variants) but I do not believe that the EPIC array is comparably comprehensive and therefore struggle to believe the numbers.

***Response:*** *The DNAm arrays continue to surprise us, too! The highly accurate prediction of chronological age (to within a couple of years median absolute error), coupled with the large proportion of variance in lifestyle factors (e.g., smoking pack years: 56% of the variance explained; alcohol consumption and BMI: >12% of the variance explained in both traits) have been reported extensively. As we allude to in the discussion, this is likely to be due to DNAm directly tracking environmental influences.*

2) Does it make sense that blood based DNA methylation profiles would capture such a large proportion of variation of arguably a brain trait? Is it feasible that a blood based biomarker would ever be a sufficiently precise predictor (particularly given the statistics presented for a training sample of 100,000 where R2 = 29%).

***Response:*** *This is an excellent point. Given the association between cognitive ability and so many health outcomes, the DNAm score is likely picking up many of these environmental components. We see this through newly reported results highlighting the attenuation of the incremental R2 value in LBC1936 (from 3.4% to 0.8%) when we include the 10 health and lifestyle traits in a very saturated null model. However, the EpiScore still remains a significant predictor (P=1.8x10^-3^).*

*Results: Paragraph 7:*

*“A multiple regression model in LBC1936 with all covariates as predictors of measured g is presented in* ***Additional File 1, Table S11****. Upon addition of the EpiScore to the model, the incremental R^2^ estimate was 0.8%.”*

*Discussion: Paragraph 4:*

*“The EpiScore can therefore reflect consequences of processes linked to cognitive health. This is evident by the attenuation of the incremental R^2^ estimate from 3.4% to 0.8% in a multiple regression model that included age and sex plus 10 covariates that are established correlates/risk factors for cognitive ability and cognitive decline. The correlation with these risk factors and the independent contribution of the EpiScore from the polygenic score for prediction of measured g emphasises the environmental variance being captured by the EpiScore.”*

*Although we cannot tell if our EpiScore is tracking processes in the brain, it does capture similar associations (to measured cognitive ability) with a variety of brain (MRI) and blood (protein) phenotypes.*

*We agree that our theoretical estimates should be treated with caution. We have edited this section accordingly (see below). We* *are in the process of doubling the GS sample size with DNAm to ~19k so will get better estimation of the DNAm architecture of g and many other traits in the near future. Furthermore, one of the paper’s co-authors (Elliot Tucker-Drob) has applied the predictor to >1,000 teenage participants in the Texas Twins Study, where it explains >11% of the variance in Maths ability [1].*

*Results: Paragraph 6:*

*“To see how the size of the training sample might affect the proportion of variance in g we could explain we simulated several scenarios. Based on a training sample size of 10,000 and an (arbitrary) assumption of 100,000 CpGs affecting the trait, with a DNAm variance components estimate of 41.6%, we would expect a DNAm prediction R^2^ value of about 4.0% (following Formula 1 from [13]).”*

3) Of the ~840,000 sites on the EPIC array, the majority of these do not vary (at least within a tissue/cell type) and are effectively 0 or 100% methylated. These are unlikely to be truly associated to the outcome, are estimated more imprecisely and more likely to result in false positive associations (due to smaller variance). How does the result change if these are excluded? What is the value of retaining these in the analysis?

***Response:*** *This is another excellent point. There is likely to be plenty of redundancy on the array through relatively invariant CpGs and (for our current purpose) sites under genetic control. We are currently working on creating a subset of probes that exclude invariant CpGs or those with a mQTL to see if we can obtain similar predictive capacity as the full array. However, with the approach presented here, we can hopefully be sure that we’re not missing anything, especially when this is the largest single cohort study to be analysed. To investigate this further, we have plotted the distribution of variances of all CpGs with PIP>0.05 versus all other probes There is little difference between average variances for each probe group, which suggests that invariant probes are not preferentially entering the model (figure below – mean variance of methylation M-values for top CpGs = 0.29 versus 0.31 for all other CpGs in Set 1; 0.32 vs 0.30 in Set 2).*

4) When the analysis concludes that "The majority of the variance was accounted for by CpG sites with small effects" is this interpretation limited to the sites directly measured by the array, or does it tell us about sites not assayed.

***Response:*** *This statement relates to CpG sites assessed on the array. We have clarified this as follows:*

Results: Paragraph 2:

“The majority of the variance explained by the DNAm array was accounted for by CpG sites assigned to the mixture with small effects”

5) Given the potential of reverse causality in epigenetic epidemiology, how should one interpret these results? Surely, these are not all causal sites. If they are not, are they consequences or result from confounders? Do these options not violate the assumptions of the statistics? Here I am questioning whether GWAS methods can simply be lifted and applied to EWAS data without the interpretation being given adequate thought.

***Response:*** *We agree that confounding could have impacted the EpiScore. We have tried to discuss this carefully and have now added the following text to emphasise these points.*

*Discussion: Paragraph 4:*

*“The EpiScore can therefore reflect consequences of processes linked to cognitive health. This is evident by the attenuation of the incremental R^2^ estimate from 3.4% to 0.8% in a multiple regression model that included age and sex plus 10 covariates that are established correlates/risk factors for cognitive ability and cognitive decline. The correlation with these risk factors and the independent contribution of the EpiScore from the polygenic score for prediction of measured g emphasises the environmental variance being captured by the EpiScore.”*

*This does not invalidate our statistical approach. At one level, statistical prediction is just that – interpretation of the features selected for the prediction requires much greater nuance, particularly for DNAm, which could be cause or more likely consequence of cognitive function or a related trait. We have added the following text to the discussion to discuss future experiments that would help to validate the predictive utility of the EpiScore*

*Discussion: Paragraph*

*“Future studies should investigate prospective associations of the EpiScore relative to measured g. Even if the former is capturing processes related to or downstream of cognitive function, it may still provide information for assessing risk of neurodegeneration.”*

*Moreover, the limitation noted is applicable to all downstream (non-genetic) omics data studies.*

6) The lack of shared signal between the genetic and epigenetic components is particularly interesting. Could these be interpreted as the EpiScore reflecting more environmental risk factors of cognition? Does this EpiScore correlate with EpiScore for smoking, alcohol or obesity with the author's state in the introduction are risk factors for cognitive decline.

***Response:*** *As mentioned in response to previous points, we have edited the manuscript to acknowledge the environmental contributions to the EpiScore. In relation to the second point, we have also looked at the correlation between the cognitive EpiScore and EpiScores for alcohol, BMI, and smoking (all derived from a previous analysis in GS and applied to LBC1936) [2]. We did not observe a relationship between the EpiScore for general cognitive ability and that for smoking (r=-0.04; p=0.26) or BMI (r=-0.005, P=0.88). However, there was a significant albeit small positive correlation between the EpiScore for g and that for alcohol consumption (R=0.12; p=4.4x10^-4^). In addition to this possibly reflecting shared environmental influences, it could also reflect a gene-environment correlation i.e., not a GxE interaction, but rather that certain genotypes favour, or are present in certain environments.*

7) Given the broad age range in the dataset, there is a huge variation in where the individuals are on their lifelong cognitive trajectory. For some participants their g score will reflect their peak cognitive state, other older individual may be undergoing cognitive decline albeit not to clinical levels. What I am alluding to here is heterogeneity in the definition of the phenotype and risk factors that underlie an individual's cognitive state at that stage of their life. How might this influence the analysis?

***Response:*** *This is another interesting point. As we mentioned above, the EpiScore has since been applied to a teenage cohort in Texas where it explained >11% of the variance in Maths ability. Further replication across different populations and refining the predictor with the next release of DNAm data from GS is of great interest to our group. We may also have sufficient numbers to stratify by age range when developing future predictors, given that individuals of different age groups will be at different points in lifecourse cognitive trajectories. We have added the following text to the discussion to acknowledge the need for additional replication across diverse populations:*

*Discussion: Paragraph 3:*

*“Nonetheless, replication of our findings in cohorts of different ages and backgrounds, and at different stages of neuro-development/degeneration will help to refine and generalise the estimates presented here.”*

8) In this statement:
Based on a training sample size of 10,000 and assuming 100,000 CpGs affect the trait, with a DNAm variance components estimate of 41.6%, we would expect a DNAm prediction R2 value of about 4.0% (following Formula 1 from [13]).
Where does the number of 100,000 CpGs come from? Are the authors suggesting that because these statistics fit the pattern observed data that is the total number of CpGs associated with cognitive function? The fact that in what is a very large sample by EWAS standards they only report three is somewhat disappointing.

***Response:*** *As mentioned in response to a previous point, the 100,000 CpGs is an arbitrary number. We have clarified this in the text*

*Results: Paragraph 6:*

*“To see how the size of the training sample might affect the proportion of variance in g we could explain we simulated several scenarios. Based on a training sample size of 10,000 and an (arbitrary) assumption of 100,000 CpGs affecting the trait, with a DNAm variance components estimate of 41.6%, we would expect a DNAm prediction R^2^ value of about 4.0% (following Formula 1 from [13]).”*

*This may be an over- or under-estimate! As mentioned above, the predictor explained >11% of the variance in Maths ability in a teenage cohort.*

*That we observed 3 CpGs to have genome-wide significant associations with g is perhaps disappointing. However, given the output from the variance components analysis where 93% ((31.3 + 7.3) / 41.6 – numbers from* ***Table S4****) of the variance explained in g was due to CpGs assigned to the mixtures with 0.1% or 0.01% variance explained, we likely need far greater numbers to reliably detect individual CpG sites. This reflects the early GWAS literature, which also had limited findings with comparably small sample sizes. With the collection of biobank scale data, it has now been shown that the genetic basis of complex traits is highly polygenic. We provide evidence that methylation associations are likely similar*.

9) How do we interpret the epiScore for cognition given it potentially captures some of the risk factors influencing cognitive function? Indeed the authors note these themselves in the Introduction. "However, blood-based 52 DNA methylation predictors of cognitive function itself, rather than its known correlates, may 53 index a wider range of risk factors for neurodegeneration." Can we then really call it a biomarker of cognition?

***Response:*** *Please see our responses to points 5 and 6 for details. Given that the EpiScore associations with the various outcomes studied here reflect those of measured g, the score seems to reflect at least part of what is being assessed via the pen and paper cognitive tests.*

10) What does it mean that all the three lead cognition CpGs are associated with age? I find this a little concerning given the age range in your cohort. I know you regressed age out, and looked the addition of an age squared term, but if the relationship between cognition and age is non-linear, then the residuals will still be associated with age. Bias may also be introduced if there are outliers in either the g or the age distributions.

***Response:*** *We appreciate the reviewer’s concerns here. However, a large proportion of sites on the Illumina array have previously been associated with chronological age, many of which have then been annotated in the EWAS catalog. In a previous Generation Scotland study, we showed that >150,000 CpGs on the EPIC array were associated with chronological age using a very conservative threshold of P<3.6x10^-8^ for both discovery and replication cohorts. Here, we also projected our EpiScore for g into two independent birth cohorts (i.e. with minimal age variation; SD < 1 year). However, we have added the following text to the discussion to note how future studies could examine nonlinear age effects on cognitive function:*

*Discussion: Paragraph 3:*

*“Whereas a near unit correlation was observed between the cognitive score residuals with and without a quadratic age term, modelling non-linear relationships may also aid locus discovery and improve external predictions.”*

*Regarding outliers, we applied a cut-off of 3.5 standard deviations from the mean for each cognitive trait (Methods: Cognitive Phenotypes in Generation Scotland) paragraph). There were no obvious age outliers in the dataset (density plot of age below).*

11) The SNP based heritability is reported as 37.9% with a 95% credible interval of [18,3% - 25.9%] how can the estimate not be included within the credible interval? Assuming the credible interval is correct, and the estimate is a typo, the precision for the SNP heritability is tighter than for the epigenetic estimate. Why is this? I assume they were performed on the same sample size.

***Response:*** *Thank you for highlighting this. It is a typo and should read 52.9 for the upper limit. We have corrected this in the updated manuscript.*

12) I disagree that the estimates from the linear mixed models in whole cohort vs the single batch are similar, one is almost 50% bigger than the other. Surely, you would expect them to be within the SE (or ~2xSE). Does this suggest some bias/differences in the datasets? What's more, what units are the SE? Are they %, if so they are tiny suggesting the estimates are extremely accurate and further highlighting how different the estimates are.

***Response:*** *The SEs for the estimates are on the same scale as the means (both can range between 0-1). We have plotted the estimates and 95% CIs for the two OSCA analyses below to highlight how similar the associations are.*

13) I couldn't find anywhere in the manuscript for the 2 LBC cohorts the age at which cognition was assessed.

***Response:*** *We have added a table of demographics for the LBC studies (****Additional File 1, Table S8****). We have also clarified this in the methods.*

*Methods: Cognitive Phenotypes in the Lothian Birth Cohorts:*

*In LBC1936 at Wave 1 (mean age 70 years), general cognitive ability was defined as the first unrotated principal component (that accounted for 52% of the variance) from a PCA of six cognitive tests from the Wechsler Adult Intelligence Scale-III UK (matrix reasoning, letter number sequencing, block design, symbol search, digit symbol, and digit span backward) [5] plus a test of vocabulary (National Adult Reading Test) [19].*

*A similar approach was taken in LBC1921 at Wave 1 (mean age 79 years), where we considered the first unrotated principal component (that also accounted for 51% of the variance) from a PCA of four cognitive tests: Raven’s Standard Progressive Matrices [20], letter-number sequencing [5], digit symbol coding [5], and the National Adult Reading Test) [19]. The cognitive tests were completed at the same visit that blood was drawn for DNA profiling in both LBC studies.”*

14) In table S2 what is the difference between g and gf?

***Response:*** *gf is a measure of fluid cognitive ability that doesn’t include the test of crystallised cognitive ability. We have clarified this in the* ***Table S2*** *legend – this information is also presented in the methods.*

*Methods: Cognitive Phenotypes in Generation Scotland:*

*“General fluid cognitive ability (g_f_) was calculated from the first unrotated principal component of logical memory, verbal fluency, and digit symbol tests. General cognitive ability (g) was derived from the first unrotated principal component from the same variables plus vocabulary.”*

Reviewer #3:
The authors conducted analysis to understand associations between cognitive function and blood-based methylation measures using a large cohort of European ancestries. The biggest issue here is, this paper feels like a big data dump and need work in carefully laying out the rationale behind the conducted work.

1.      The description of the "Statistical analysis" in the supplement file 3 is not organized not is it very clear. There is no rationale under any of the analysis done. The paper feels like a big data dump. The authors may want to have several sub-sections to summarize different types of analysis first with each sub-section having clear rationale behind. The authors can then provide details of the analyses within each sub-section. It is currently all blended together with no rationale in one gigantic paragraph of about 5 pages long.

***Response:*** *We apologise for any lack of clarity in our methods section. We have made major edits to this section with sub-section headings for each analysis. We have also edited the main narrative in the Results section to help the reader understand what we are analysing and why we are doing this.*

2.      There should also be details on the cohort in Online Methods instead of referring readers to several publications. What is the timeline of cognitive function measures and DNA methylation samples? Concurrent?

***Response:*** *We agree with the reviewer and have added the following details to the methods section to introduce the GS cohort.*

*Methods: The Generation Scotland Cohort*

*“Details of the Generation Scotland: Scottish Family Health Study (GS) have been described in detail elsewhere [1, 2]. Briefly, GS comprises over 20,000 individuals comprehensively profiled for genetic, clinical, lifestyle, and sociodemographic data. Around 8,000 participants aged between 35 and 65 years were initially recruited through GP surgeries from five regions across Scotland. These participants were then asked to invite family members to join the study. Recruitment took place between 2006 and 2011 and the structure included relatives from up to three generations per family (around 5,600 families participating in total). The age range of the cohort at the study baseline was 18 to 99 years. A subset of 9,162 individuals from GS (aged 18 to 93 years, mean=49.7, SD=13.6) had genome-wide DNA methylation measured [3]. This subset was processed in two batches, hereafter referred to as “Set 1” and “Set 2”.”*

*We have added Supplementary tables (****Table S8****) for the two LBC studies to show the demographic and health/disease variable summary statistics. The timeline of the cognitive function and DNAm measures are concurrent as we now emphasise in the Results and Methods sections*

*Results: Paragraph 1:*

*“Blood-based DNA methylation and general cognitive ability (g) were assessed concurrently in 9,162 adult participants from the Generation Scotland cohort [8, 9]”*

*Results: Paragraph 5:*

*“These weights were applied to CpGs in two independent studies, The Lothian Birth Cohort 1936 (LBC1936) and The Lothian Birth Cohort 1921 (LBC1921; n=844 and n=427 with concurrently measured DNAm, cognitive scores, and cognitive polygenic scores available, respectively;* ***Additional File 2, Figure S3; Additional File 3, Methods****).”*

*3*.      The samples are from two sets "5,200 related individuals from Set 1, and 4,588 genetically unrelated individuals from Set 2". It is important to clarify 1) what kind of relatedness are in Set 1, 2) if analyses using Set 1 have taken care of the relatedness. These should be clarified in "Statistical analysis".

***Response:*** *We thank the reviewer for highlighting this. As mentioned in our response to point 2, we have a complex family-based structure in the Generation Scotland study. We control for this relatedness using multiple approaches. First, we simultaneously fit SNP and DNAm data in a BayesR+ model, whereby the effect of each SNP or CpG are fit conditional on all other variables and their covariance structure. This controls for genetic relatedness [3, 4]. Secondly, we use a different methodology (OSCA) where epigenetic similarity matrices are fitted. As the Set 2 data contain genetically unrelated individuals to begin with, these estimates are completely independent of family structure. We have updated the methods section to provide a description of the OSCA software.*

*Methods: Sensitivity Analysis:*

*“Sensitivity analyses were performed using OSCA to estimate the proportion of variance explained in cognitive tests by epigenetic data. OSCA is a software tool designed for the analysis of complex traits using multiple omics data types, including genome-wide DNAm data. Omics relationship matrices (ORMs) were estimated using all probes to determine inter-individual relationships, analogous to a genomic relationship matrix (GRM), which is used to estimate SNP-based heritability. ORMs were computed separately using DNAm data from Generation Scotland Set 1 (related individuals) and Set 2 (unrelated individuals). Each ORM was subsequently fitted to a mixed linear model to estimate the variance explained by all DNAm probes using the restricted maximum likelihood (REML) method.”*

4.      Please provide details on how "predicted epigenetic scores" were obtained.

***Response:*** *We thank the reviewer for raising this point and agree that there was insufficient detail in the previous version of the manuscript. We have added the following details to describe how the EpiScores were obtained:*

*Results: Paragraph 5:*

*“A weighted linear Epigenetic Score (EpiScore) for g that included all CpG sites in the EWAS was applied to two independent cohorts to determine the proportion of variance in g that can be explained by a single predictor variable. The weights for each CpG were the mean posterior effect sizes from the EWAS model of g. These weights were applied to CpGs in two independent studies, The Lothian Birth Cohort 1936 (LBC1936) and The Lothian Birth Cohort 1921”*

5.      For QC steps, please provide number of CpGs removed at each step and number of subjects removed at each step clearly.

***Response:*** *We apologise to the reviewer for the lack of detail in this section. We have added further information to the methods section describing the DNA methylation processing steps for Generation Scotland and Lothian Birth Cohort datasets. This information includes pre- and post-QC sample and probe counts, along with samples omitted at each stage of the quality control process. This can be found in the “****Methylation preparation in Generation Scotland****” and “****Methylation preparation in the Lothian Birth Cohorts****” subsections:*

*Methylation preparation in Generation Scotland*

*“Three Set 1 individuals who had answered “yes” to presence of all of 16 self-reported disease conditions in the study’s health questionnaire were excluded from the analysis. Filtering for outliers (N_Set1_=8062; N_Set2_=83), sex mismatches (N_Set1_=19; N_Set2_=12), non-blood samples (N_Set1_=13), and poorly detected samples was performed (N_Set1_=18;) [21]. Further filtering was then carried out to remove CpGs with missing values, non-autosomal and non-CpG sites (N=22,163), CpGs with missing values and poorly-detected probes (N_Set1_=5,910; N_Set2_=8,878). Five individuals with a self-reported diagnosis of Alzheimer’s disease were removed, along with samples with missing covariate information or cognitive variables. Following sample filtering, 9,162 complete cases remained comprising 4,901 Set 1 individuals and 4,261 Set 2 individuals, with 764,525 probes in common between the two.”*

*Methylation preparation in the Lothian Birth Cohorts*

*“We considered blood-based DNA methylation data from the age 70 (LBC1936) and age 79 (LBC1921) samples. Methylation was assessed on the Illumina 450k array – the predecessor of the EPIC array. Processing and quality control have been described previously [1, 28, 29]. This included steps to remove methylation samples and individuals with poor quality control measures, along with individuals who had mismatching genotypes or predicted sex information. DNA methylation was measured at three time points (set 1, set 2 and set 3) and comprised 2,195, 996 and 552 samples, respectively. Prior to quality control, each set had 485,512 probes. Twenty-three duplicate samples were removed from set 2. Set 1 and set 2 had 123 duplicates between them and a sample was removed from each duplicate pair (108 from set 1, 15 from set 2). Sets 1 and 2 were then combined (set12). Ten duplicates were removed from set 3. There were also 31 duplicates between set 3 and set 12. 26 samples were removed from set 3 and five were removed from set12. The three sets were combined (set123) and comprised 3,556 samples. Samples and probes were filtered on low call rates (fractions of probes with a p-value smaller than 0.01), with a threshold of 95% for both samples and probes. 3,525 samples and 470,278 probes remained after this step. Finally, sex chromosome probes were removed, leaving a final dataset comprising 459,309 probes and 3,525 samples. The current study used a subset from this dataset comprising 381,846 CpGs (overlapping with those included in the Generation Scotland analyses) for 861 LBC1936 (436 LBC1921) individuals – 34 LBC1936 individuals were excluded due to DNAm being assessed as part of a separate analysis batch.”*

6.      Much of the description of the statistical analysis is not clear, for example, the following description of analysis is not clear: "Linear regression was used to test for an association between the predicted epigenetic score (predictor) and measured general cognitive ability (outcome), in models adjusting for age, sex, and the polygenic score. Age- and sex-adjusted linear regression models were used to relate the Epigenetic Score (EpiScore) for g and the measured g score (predictors) with common risk factors (outcomes) for cognitive decline, frailty, and dementia: body mass index (BMI in kg/m2); year of education; self-reported smoking (ever versus never); self-reported weekly units of alcohol; self-reported high blood pressure (yes/no); lung function (forced expiratory volume in one second) adjusted for age, sex, and height; time taken to walk six metres (seconds); socioeconomic deprivation (Scottish Index of Multiple Deprivation in LBC1936 and social grades based on
highest reached occupation [21] in LBC1921); and depression (HADS-D total from the Hospital Anxiety and Depression questionnaire) [22, 23, 24]. BMI, six metre walk time, and units of alcohol were log transformed to reduce skew - a constant of one was added to the alcohol units prior to transforming." Please rewrite these sentences. It is not clear what has been done.

***Response:*** *As requested, we have rewritten this section. We hope that the updated version provides a clearer description of the analyses undertaken.*

*Methods: EpiScore and measured g associations with common risk factors in the Lothian Birth Cohorts:*

*“Age- and sex-adjusted linear regression models were used to relate the Epigenetic Score (EpiScore) for g and the measured g score (predictors) with common risk factors (outcomes) for cognitive decline, frailty, and dementia. Each outcome was modelled independently in a separate regression model. The outcomes studied were : body mass index (BMI in kg/m2); year of education; self-reported smoking (ever versus never); self-reported weekly units of alcohol; self-reported high blood pressure (yes/no); lung function (forced expiratory volume in one second) adjusted for age, sex, and height; time taken to walk six metres (seconds); socioeconomic deprivation (Scottish Index of Multiple Deprivation in LBC1936 and social grades based on highest reached occupation [37] in LBC1921); and depression (HADS-D total from the Hospital Anxiety and Depression questionnaire) [26, 38, 39]. Prior to the analyses, BMI, six metre walk time, and units of alcohol were log transformed to reduce skew – a constant of one was added to the alcohol units before the log transformation.”*

7.      When using BayesR+ package, it is important to also describe statistical methods used instead of just referring to "the package has been used".

***Response:*** *We agree that it is important to describe the statistical methods fully. We have restructured the manuscript, moving much of the online methods to the main document, and added extensive details of the BayesR+ methodology to the new Methods section of the manuscript.*

*Methods: Variance components analysis and EWAS using BayesR+:*

*“BayesR+ was used for the EWAS and to estimate the proportion of variance in cognitive traits explained by genetics and DNA methylation. BayesR+ is a software implemented in C++ for performing Bayesian penalised regression and Gaussian mixture-based variance partitioning on complex traits [8]. The joint and conditional effects of methylation sites (n=764,525) on cognitive traits were examined. Phenotypic and methylation data were scaled to mean zero and unit variance. The prior distribution comprised a series of Gaussian distributions which corresponded to effect sizes of different magnitudes (i.e. methylation sites with small, medium and large effect sizes), as well as a discrete spike at zero which allows for the omission of probes with non-identifiable effects. The prior mixture variances were set to 0.0001, 0.001 and 0.01. Phenotypes were corrected for age, sex, BMI and epigenetic smoking score [9], and the DNA methylation data were corrected for batch, age, sex and epigenetic smoking. Adding a quadratic term for age in the correction of cognitive phenotypes made negligible differences to the trait residuals (r>0.98 between linear model residuals for each trait).*

*Methods: Estimating the variance components and individual CpG effects:*

*To obtain estimates of variance accounted for in cognitive traits by methylation data and individual CpG associations with the cognitive test scores, Gibbs sampling was performed to sample over the posterior distribution conditional on the input data. The Gibbs algorithm consisted of 10,000 samples and 5,000 samples of burn-in after which a thinning of 5 samples was applied to reduce autocorrelation. The process was repeated over four chains, initializing a different random number seed for each chain. The last 250 iterations from each chain were combined for downstream analyses. For the EWAS, CpGs within 2.5kb and highly correlated (absolute Pearson correlation >0.5) with a lead CpG with posterior inclusion probability greater than 0.2 were grouped together. For each probe group, we calculated the proportion of iterations for which at least one probe was included in the model, yielding the group posterior inclusion probability. We then calculated the average (across the 1,000 iterations) sum of the squared regression coefficients for the probe group to give the contribution of the group to the total variance. Finally, we highlighted the lead CpG for the groups where the combined posterior inclusion probability was >0.80.”*

8.      Please describe in details the statistical method with steps and data used to generate each table, for example, for Table S2, it is currently described as "Variance components analyses indicated that 41.6% [95% Credible Interval 31.0%, 53.0%] of variance in g was explained by all DNA methylation probes". Please describe clearly 1) statistical approach and steps, 2) data that generated these proportions and the confidence interval.

***Response:*** *We have updated the methods section of the manuscript to further clarify and describe how these estimates were obtained.*

*Methods: Estimating the variance components and individual CpG effects:*

*“The variance components estimates are taken as the mean sum of squared standardised mean posterior effect sizes across the 1,000 iterations with the 2.5%ile and 97.5%ile forming the 95% credible interval.”*

*Results: Paragraph 2*

*Of the prior distributions specified in the BayesR+ analysis. the majority of the variance explained by the DNAm array was accounted for by CpG sites assigned to the mixture with small effects; three prior mixture distributions were set with small (0.01%), medium (0.1%), and large (1%) variances for effect sizes (0.01%; Additional File 1, Table S4).*

*Results: Paragraph 4*

*The BayesR+ method also allowed us to investigate the associations between g and individual CpG sites, which were assigned to one of the three mixtures. We identified three unique lead DNAm sites with a posterior inclusion probability (PIP) greater than 0.80 and, after accounting for highly correlated CpG clusters, a group-based PIP>0.95 (Additional File 2, Figure S2; Additional File 1, Table S5; the entire output is available at https://gitlab.com/danielmccartney/ewas_of_cognitive_function).*

9.      There are many places that were described loosely, for example, in the following sentence "sensitivity analysis using a linear mixed model approach [5] with genetic and epigenetic relationship matrices yielded near identical results (43.4% (SE 0.03));", it is not clear what "genetic and epigenetic relationship matrices" are, and how the matrices are used to generate "identical results", and what is the aim of this sensitivity analysis.

***Response:*** *We thank the reviewer for pointing this out. In the updated manuscript, we provide extensive details on the methodology for these sensitivity analyses. We also note that these analyses were undertaken to confirm that our results are not a function of the analysis method used. Please also note we used epigenetic relationship matrices in the OSCA sensitivity analysis, not genetic and epigenetic matrices, as listed in error in the submitted version. This has been corrected in the main text.*

*Methods: Sensitivity Analyses:*

*“Sensitivity analyses were performed using OSCA to estimate the proportion of variance explained in cognitive tests by epigenetic data. OSCA is a software tool designed for the analysis of complex traits using multiple omics data types, including genome-wide DNAm data. Omics relationship matrices (ORMs) were estimated using all probes to determine inter-individual relationships, analogous to a genomic relationship matrix (GRM), which is used to estimate SNP-based heritability. ORMs were computed separately using DNAm data from Generation Scotland Set 1 (related individuals) and Set 2 (unrelated individuals). Each ORM was subsequently fitted to a mixed linear model to estimate the variance explained by all DNAm probes using the restricted maximum likelihood (REML) method. These analyses were undertaken to confirm that the results obtained were not a function of the analysis method used.”*

***References***

1. *Raffington, L., Tanksley, P. T., Sabhlok, A., Vinnik, L., Mallard, T., King, L. S., Goosby, B., Harden, K. P., & Tucker-Drob, E. M. (2021). Socially stratified epigenetic profiles are associated with cognitive functioning in children and adolescents. BioRxiv, 2021.08.19.456979. https://doi.org/10.1101/2021.08.19.456979*
2. *McCartney, D. L., Hillary, R. F., Stevenson, A. J., Ritchie, S. J., Walker, R. M., Zhang, Q., Morris, S. W., Bermingham, M. L., Campbell, A., Murray, A. D., Whalley, H. C., Gale, C. R., Porteous, D. J., Haley, C. S., McRae, A. F., Wray, N. R., Visscher, P. M., McIntosh, A. M., Evans, K. L., … Marioni, R. E. (2018). Epigenetic prediction of complex traits and death. Genome Biology. https://doi.org/10.1186/s13059-018-1514-1*
3. *Trejo Banos, Daniel, Daniel L. McCartney, Marion Patxot, Lucas Anchieri, Thomas Battram, Colette Christiansen, Ricardo Costeira, et al. 2020. “Bayesian Reassessment of the Epigenetic Architecture of Complex Traits.” Nature Communications.* [*https://doi.org/10.1038/s41467-020-16520-1*](https://doi.org/10.1038/s41467-020-16520-1)*.*
4. *Patxot, M., Banos, D. T., Kousathanas, A., Orliac, E. J., Ojavee, S. E., Moser, G., Sidorenko, J., Kutalik, Z., Mägi, R., Visscher, P. M., Rönnegård, L., & Robinson, M. R. (in press). Probabilistic inference of the genetic architecture of functional enrichment of complex traits. Nature Communications. MedRxiv, 2020.09.04.20188433. https://doi.org/10.1101/2020.09.04.20188433*

**Second round of review**

**Reviewer 1**

I thank the authors for conducting the additional analyses, and for answering all my questions satisfactorily. Proving the additional information and re-structuring the Methods section helped a lot in understanding the outstanding work provided in this manuscript. In addition, I like the proposed labelling of Figure 1 panels C and D, and have no further questions or concerns.

**Reviewer 2**

The manuscript is improved by the addition of the methodological details. I still think more narrative on what biological question each analysis seeks to address, and interpretation of the results in this context would be beneficial to the reader. There are still a number of analyses with limited discussion. Aligning the results to specific research questions would increase the value of the paper's findings. More concerning to me, is that in their response the authors attribute a worrying number of reviewers concerns to errors in their original manuscript (incorrect protein name, incorrect probe ID,…). Although these appear to generally be typos, it raises the question as to whether, there are more pervasive errors in the manuscript that haven't been picked up as they did not align with the reviewer's comments. Not to mention that in the response document, there were errors with the references - indicating a lack of attention to detail. Are the authors confident their analysis and manuscript are now completely correct?

**Authors Response**

**Point-by-point responses to the reviewers’ comments:**

*Please find attached a resubmission of the above-quoted manuscript in which we have taken steps to address the referees’ points. We have also applied formatting changes requested by the editor, provided DOIs/references for CpG weights and analysis code, and modified the abstract as suggested. A more detailed response to the referees’ comments is provided below.

Please note a change to Table S10 in which smoking and hypertension models have been replaced with linear regression outputs. This is to maintain consistency with the values presented in Figure 1. Logistic regression outputs for these models are presented in the footnote accompanying the table.*


Reviewer reports:

Reviewer #1: I thank the authors for conducting the additional analyses, and for answering all my questions satisfactorily. Proving the additional information and re-structuring the Methods section helped a lot in understanding the outstanding work provided in this manuscript. In addition, I like the proposed labelling of Figure 1 panels C and D, and have no further questions or concerns.

***Response****: Thank you very much!*


Reviewer #2: The manuscript is improved by the addition of the methodological details. I still think more narrative on what biological question each analysis seeks to address, and interpretation of the results in this context would be beneficial to the reader. There are still a number of analyses with limited discussion. Aligning the results to specific research questions would increase the value of the paper's findings. More concerning to me, is that in their response the authors attribute a worrying number of reviewers concerns to errors in their original manuscript (incorrect protein name, incorrect probe ID,…). Although these appear to generally be typos, it raises the question as to whether, there are more pervasive errors in the manuscript that haven't been picked up as they did not align with the reviewer's comments. Not to mention that in the response document, there were errors with the references - indicating a lack of attention to detail. Are the authors confident their analysis and manuscript are now completely correct?

***Response:*** *Thank you for your comments. The manuscript has now been updated to include rationale behind the analyses performed which we hope will assist with the narrative. Results have been divided with subheadings to highlight each question, which we hope will improve flow and readability. Moreover, we have expanded the discussion to highlight of potential applications of the EpiScore, as an aid to predict risk of neurodegeneration. These additions are highlighted in yellow. Regarding the concerns about errors, we accept these points and have since ensured all code, references, and reported results are in agreement.*


Reviewer #3: The authors have greatly improved the presentation of the paper. Methods are now clearly described. However, the rationale underlying each analysis is still missing. The meaning of the findings are not very clear either. The authors mentioned at the end "BayesR+ will clearly lead to both locus discovery and more accurate DNAm-based predictors of cognitive function". Is this paper help future development of a prediction model for cognitive function using methylation measure

***Response****: Thank you for your comments. Each analysis has been allocated a subheading in the results section and further information describing their rationale has been added. Further interpretation has been added to the discussion, where we highlight the associations between the EpiScore and brain health/lifestyle patterns, and its potential as a predictor of brain health and neurodegeneration. These new additions are highlighted in yellow.

For clarity, we have rephrased the discussion to read

“increasing EWAS sample sizes will likely lead to both locus discovery and more accurate DNAm-based predictors of cognitive function”

The take-home message of this point is that larger sample sizes are likely to improve prediction, as we have reported our estimates to comprise CpGs of small effects. We have therefore removed the reference to the method used.*
